# Ninjurin-1 mediates cell lysis and detrimental inflammation of PANoptosis during influenza A virus infection

**DOI:** 10.1038/s41392-025-02391-9

**Published:** 2025-09-23

**Authors:** Yitian Xu, Ying Zheng, Yan Liu, Cheng Wei, Juan Ren, Wenting Zuo, Runqing Gu, Hongyu Liu, Xiaoyan Deng, Yaxue Liu, Xiao Shang, Weiwei Ge, Ziyao Li, Yijiao Huang, Di He, Xuhui Shen, Zeyi Wang, Chen Lyu, Zai Wang, Yuxian Mu, Zihuan Zhang, Hongping Wu, Haibo Li, Bin Cao

**Affiliations:** 1 China-Japan Friendship Hospital (Institute of Clinical Medical Sciences), Chinese Academy of Medical Sciences & Peking Union Medical College, Beijing, China; 2 National Center for Respiratory Medicine; State Key Laboratory of Respiratory Health and Multimorbidity; National Clinical Research Center for Respiratory Diseases; Institute of Respiratory Medicine, Chinese Academy of Medical Sciences; Beijing Key Laboratory of Surveillance, Early Warning and Pathogen Research on Emerging Infectious Diseases; New Cornerstone Science Laboratory; Department of Pulmonary and Critical Care Medicine, Center of Respiratory Medicine, China-Japan Friendship Hospital, Beijing, China; 3 Department of Pulmonary and Critical Care Medicine, China-Japan Friendship Hospital, Capital Medical University, Beijing, China; 4 Department of Critical Care Medicine, Yantai Affiliated Hospital of Binzhou Medical University, Yantai, Shandong China; 5 Department of Pulmonary and Critical Care Medicine, The Second Affiliated Hospital of Harbin Medical University, Harbin, Heilongjiang China; 6 Medical Data and Medical Record Management Center, Huashan Hospital, Fudan University, Shanghai, China; 7 Tsinghua University-Peking University Joint Centre for Life Sciences, Tsinghua University, Beijing, China; 8 School of Basic Medical Sciences, Tsinghua University, Beijing, China

**Keywords:** Infectious diseases, Respiratory tract diseases, Infectious diseases, Innate immunity, Inflammation

## Abstract

Influenza A virus (IAV) induces ZBP1-mediated PANoptosis, a form of lytic inflammatory cell death characterized by concurrent activation of the pyroptosis, necroptosis and apoptosis pathways. Ninjurin-1 (NINJ1) is a recently identified mediator of plasma membrane rupture but functions diversely in different types of cell death. However, little is known about the role of NINJ1 in IAV-induced PANoptosis and viral pneumonia. Here, we report that IAV infection triggered an increase in the expression of NINJ1, which then oligomerized and mediated cell lysis in infected macrophages. The deficiency of NINJ1 prevented plasma membrane rupture and the release of DAMPs and IL-1β without affecting the progression of cell death. Activation of any single PANoptosis pathway was sufficient to trigger the oligomerization of NINJ1 and robust cell lysis. Accordingly, only when all PANoptosis pathways were concurrently blocked could the oligomerization of NINJ1, cell death, and cell rupture be prevented. Ablation of NINJ1 in vivo also alleviated IAV-induced lung injury and mortality. Furthermore, we revealed an association between NINJ1 upregulation and poor outcomes in patients with COVID-19. Collectively, our findings indicate a pivotal role of NINJ1 in the immunopathology of IAV infection and its potential as a bioindicator of disease severity and prognosis in viral pneumonia and viral sepsis.

## Introduction

IAV has posed a great disease burden on global health because of seasonal epidemics and sporadic pandemics. After infection, host cells undergo lytic cell death. In addition to respiratory epithelial cells—the primary target for initial viral infection and replication—macrophages are the most studied cell type in IAV-induced cell death. Since infection of macrophages is usually abortive,^1,2^ more attention has been given to the inflammatory responses induced by their death. IAV-infected macrophages undergo ZBP1-mediated PANoptosis, a lytic and inflammatory cell death modality in which the pyroptosis, necroptosis, and apoptosis pathways are activated together.^3,4^ During this process, a molecular scaffold called PANoptosome is assembled for the interactions and activation of machinery required for PANoptosis, which consists of ZBP1, RIPK3, RIPK1, Caspase-8, NLRP3, and Caspase-6.^4–6^ Like other classical lytic cell death (i.e., pyroptosis and necroptosis), PANoptosis leads to plasma membrane rupture (PMR) and the subsequent release of massive amounts of DAMPs and cytokines, which ignites robust inflammatory responses.

Although cell death and inflammation are crucial for viral clearance, it is well recognized that excessive and unchecked inflammation, rather than infection itself, is the pathophysiology of severe bacterial and viral infection. Based on this, constituents of cell death pathways seem promising targets of immunomodulatory therapeutics. However, efforts to identify ideal therapeutic targets have progressed little. To date, only MLKL and GSDMD, both of which are regarded as terminal executioners in lytic cell death, have been reported to be potential therapeutic targets,^7–10^—though our findings from this study appear to challenge this perspective. Ablation of other constituents of PANoptosis, including ZBP1, RIPK3, Caspase-6, and the NLRP3 inflammasome, fails to yield a better prognosis in IAV-infected mice.^5,7,11–16^ This reflects the dilemma of optimally regulating the cell death process to eliminate infections while preventing uncontrolled inflammation.

Ninjurin-1 (NINJ1) is a membrane protein identified as a pivotal mediator of PMR, and its underlying structural basis has been intensively studied recently.^17–21^ Although the proposed models for NINJ1-mediated PMR differ across studies—with Degen et al. suggesting that NINJ1 forms filaments that cap membrane edges, thereby mediating the leakage of large cellular contents via its hydrophilic side,^18^ David et al. proposing that NINJ1 uses its hydrophobic side to form a membrane-encasing disk which is then cut and shed,^19^ and Pourmal et al. demonstrating that dissociation of NINJ1 face-to-face dimers is prerequisite for its activation^20^—the oligomerization of NINJ1 can be regarded as the hallmark event underlying its mediation of PMR. As a result of NINJ1 oligomerization, the cellular contents, including many proinflammatory DAMPs, are released.^17^ However, NINJ1 seems dispensable for the formation of GSDMD pores and the GSDMD-dependent release of IL-1β in pyroptosis induced by activation of both canonical and noncanonical NLRP3 inflammasome.^17^ In addition to its role in pyroptosis, NINJ1 has been shown to play a role in multiple types of cell death.^17,22^ In contrast, cell lysis in MLKL-mediated necroptosis seems to be independent of NINJ1.^17,22,23^ Additionally, the requirement of NINJ1 for PMR during ferroptosis is trigger dependent.^22^ The mechanism accounting for this discrepancy is far from clear, indicating an erratic feature of NINJ1 in more complex circumstances. Given the lytic feature of PANoptosis induced by IAV, we hypothesize that NINJ1 also plays a role in this process. Actually, NINJ1 has already been reported to be a mediator of PANoptosis induced by comorbid heat stress and infection.^24^ In that model, ablation of NINJ1 rescued BMDMs from cell death and did not impair the inflammasome-dependent release of IL-1β and IL-18.^24^ Although IAV infection and heatstroke are similar in that PANoptosis induced by both are initiated by ZBP1,^25^ it is unknown whether NINJ1 functions similarly during IAV infection. In addition, it is necessary to elucidate how NINJ1 cooperates with other activated pore-forming PANoptosis executioners (i.e., GSDMD, GSDME, and MLKL) during IAV infection to regulate cell death, PMR, and the release of cytokines, especially IL-1β, which is GSDMD-dependent and NINJ1-independent in pyroptosis.^17,26^

In this study, we systematically evaluated the role of NINJ1 in the context of IAV-induced PANoptosis. We identified that the expression of NINJ1 increases following IAV infection, particularly in myeloid cells, and lethal-dose IAV infection induces a more pronounced upregulation of NINJ1 compared to non-lethal doses. Upregulated NINJ1 oligomerized and mediated detrimental cell lysis and the release of important inflammatory mediators during IAV-induced macrophage PANoptosis. Oligomerization of NINJ1 was initiated by the activation of any of the three cell death pathways involved in PANoptosis (i.e., pyroptosis, apoptosis, or necroptosis pathway). However, termination of NINJ1 oligomerization and cell lysis could be only achieved by inhibiting all the PANoptosis pathways at the same time. In addition, through in vivo comparative analysis of immunopathological phenotypes in IAV-infected wild-type versus *Ninj1*^-/-^ mice and single-cell profiling of lung tissues from IAV-infected mice and BALF samples from COVID-19 patients, we provided preliminary insight into its potential as a therapeutic target and severity biomarker in influenza and COVID-19.

## Results

### NINJ1 is upregulated upon IAV infection

**Fig. 1:**
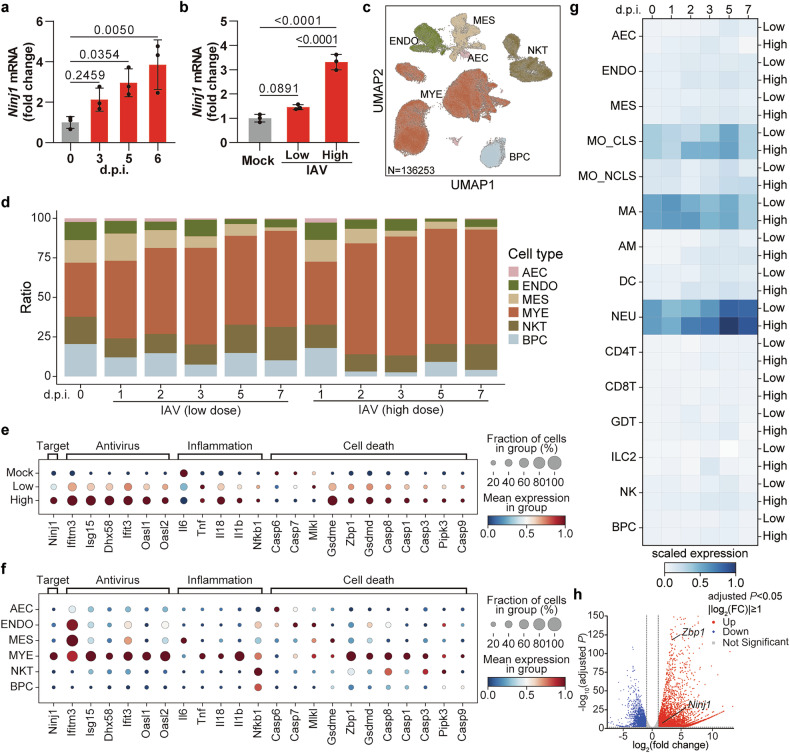
NINJ1 is upregulated upon IAV infection. **a,** **b** qRT‒PCR analysis of relative *Ninj1* expression in murine lungs at indicated dpi (1×10^5^ PFU) (**a**) or at 5 dpi infected with low (1×10^3^ PFU) or high (1×10^5^ PFU) IAV dose (**b**). **c** UMAP of 136,253 single cells from lungs at indicated dpi with low/high IAV dose. AEC, alveolar epithelial cell; ENDO, endothelial cell; MES, mesenchymal cell; MYE, myeloid cell; NKT, NK cell and T cell; BPC, B cell and plasma cell. **d** Proportion of major cell clusters by group and dpi. **e,** **f** Dot plots indicating the relative expression of indicated genes in different groups (**e**) or in different cell clusters (**f**). **g** Matrix plot of *Ninj1* expression per cell cluster at different dpi with low/high IAV dose. MO_CLS, classical monocyte; MO_NCLS, non-classical monocyte; MA, macrophage; AM, alveolar macrophage; DC, dendritic cell; NEU, neutrophil; CD4T, CD4^+^ T cell; CD8T, CD8^+^ T cell; GDT, γδ T cell; ILC2, group 2 innate lymphoid cell; NK, natural killer cell. **h** Volcano plot of 1,028 differentially expressed genes in IAV- and mock-infected BMDMs at 12 hpi. Red and blue dots represent 4847 upregulated and 2813 down-regulated genes respectively. *n* = 3 biological replicates/group. Data are representative of at least two independent experiments (**a**, **b**) and are presented as mean ± SD. Analysis was performed via one-way ANOVA (**a**, **b**)

**Fig. 2:**
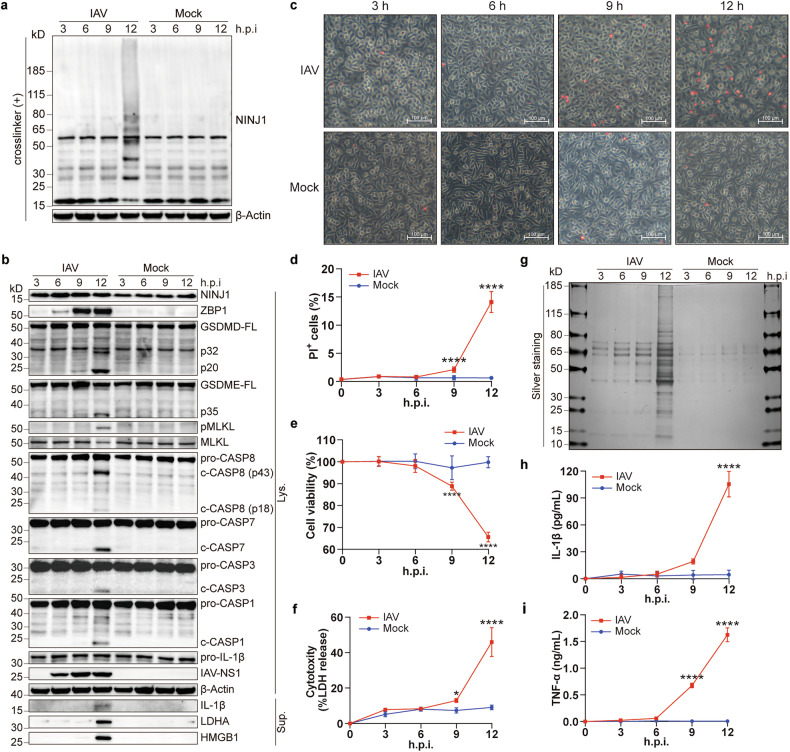
Oligomerization of NINJ1 synchronizes with PANoptosis during IAV infection. **a,** **b** Immunoblots of indicated proteins in BMDMs at indicated h.p.i. (hours post infection), BS^3^-crosslinked (**a**) or non-crosslinked (**b**). FL, full length; c-, cleaved; Lys., lysates; Sup., supernatants. **c**–**f** Representative images (**c**) and quantification (**d**) of PI^+^ cells, cell viability (**e**), and LDH release (**f**) in BMDMs treated as panel (**a,** **b**). Scale bars, 100 μm. **g** Silver staining of supernatants from (**b**). **h,** **i** Concentrations of IL-1β (**h**) and TNF-α (**i**) in supernatants from (**f**). Data are representative of three independent experiments and are presented as mean ± SD. Two-way ANOVA was used. *p < 0.05; ****p < 0.0001

Since little is known about the expression pattern of NINJ1 during viral infection, we first determined the transcript level in IAV-infected murine lungs. The expression of *Ninj1* tended to increase after infection (Fig. 1a), and the increase in expression was more pronounced when infected with a lethal dose (Fig. 1b), which indicated its possible association with disease severity. To further investigate its temporal expression pattern in different cell types, we analyzed the single-cell RNA sequence (scRNA-seq) data from murine lungs infected with a lethal dose or a non-lethal dose of IAV at different dpi produced by our group recently^27^ (CNCB, PRJCA034049; Supplementary Table 1). The cells were clustered into 6 major types and 15 subtypes (Fig. 1c–g and Supplementary Fig. 1a–d). In line with the results shown in Fig. 1b, the *Ninj1* level was elevated more after a lethal dose challenge (Fig. 1e). *Ninj1* was expressed mainly in myeloid cells (MYEs) rather than alveolar epithelial cells (AECs) (Fig. 1f), and the proportion of MYEs expanded upon IAV infection, especially in macrophages (MAs) (Fig. 1d and Supplementary Fig. 1c). Specifically, among MYEs, *Ninj1* was most highly expressed in neutrophils (NEUs), followed by MAs (Fig. 1g). Although the expression of *Ninj1* increased in most cell types, the expression peak did not synchronize. The *Ninj1* expression peaked at 1 to 2 dpi in MAs and at ~5 dpi in NEUs (Fig. 1g). Generally, a high dose of IAV resulted in higher expression of NINJ1 in most cell types (Fig. 1g). Notably, the expression patterns of numerous inflammation- and PANoptosis-associated genes (e.g., *Il1b*, *Il18*, *Zbp1*, *Gsdmd*, and *Casp8*) were similar to those of *Ninj1*, indicating *Ninj1*’s potential role in IAV-induced, ZBP1-mediated PANoptosis (Fig. 1e, f). Considering that MAs presented the second highest expression of *Ninj1* and that ZBP1-mediated PANoptosis has been well depicted in MAs, we aimed to confirm this phenomenon in BMDMs in vitro. In line with our scRNA-seq results, RNA-seq analysis of IAV-infected BMDMs (CNCB, PRJCA034048) revealed that *Ninj1* and *Zbp1* were upregulated (Fig. 1h). Additionally, this upregulation was confirmed at the protein level (Fig. 2b). Overall, our in vivo and in vitro transcriptional analyses preliminarily revealed a potential association between *Ninj1* and disease progression as well as macrophage PANoptosis during IAV infection.

### Oligomerization of NINJ1 synchronizes with PANoptosis during IAV infection

Upregulation of NINJ1 does not necessarily indicate PMR, whereas oligomerization does. NINJ1 undergoes oligomerization on the plasma membrane once activated during lytic cell death, though the exact trigger is unclear.^17,19^ Apparent NINJ1 oligomerization was detected at 12 hours post infection (hpi) (Fig. 2a). We also measured classical indicators of PANoptosis, including the activation of PANoptosis initiators and executioners, the release of LDH and inflammatory cytokines, and the percentage of PI^+^ cells. Coincidentally, the pyroptosis (cleavage of GSDMD and GSDME), apoptosis (cleavage of Caspase-3, -7, and -8), and necroptosis (phosphorylation of MLKL) pathways were also activated at 12 hpi, except for the upregulation of ZBP1 (Fig. 2b), which occurred as early as 6 hpi. At the same time, the percentage of PI^+^ cells and LDH release increased significantly as cell viability decreased (Fig. 2c–f), which are indicators of PMR and lytic cell death. Silver staining of the released proteins in the supernatants revealed no obvious selectivity for molecular weight (Fig. 2g). We noted that obvious elevation of TNF-α preceded elevation of IL-1β, which occurred at 9 hpi and 12 hpi, respectively (Fig. 2h, i), in line with the dependency of IL-1β release on cell lysis. In addition to LDH, another well-known DAMP, HMGB1, was also released in abundance (Fig. 2b). Overall, oligomerization of NINJ1 and occurrence of PANoptosis are highly synchronous. This finding prompted us to hypothesize that NINJ1 might play an important role in the course of IAV-induced PANoptosis.

### NINJ1 mediates IAV-induced cell lysis along with the release of DAMPs and IL-1β without compromising cell death

**Fig. 3:**
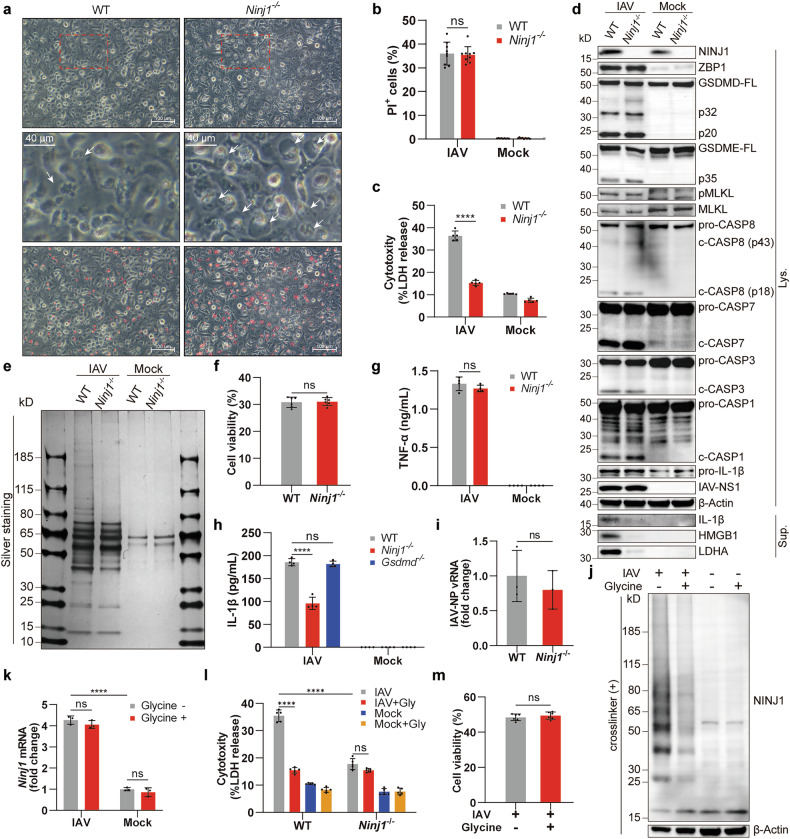
NINJ1 mediates IAV-induced cell lysis without compromising cell death. **a,** **b** Representative images (**a**) and quantification (**b**) of PI^+^ cells in wild-type (WT) and *Ninj1*^-/-^ BMDMs at 12 hpi. The arrows indicate unlysed cells exhibiting a balloon-like morphology. Scale bars, 100 μm and 40 μm in the enlarged image. **c**–**f** LDH release (**c**), immunoblots of the indicated proteins (**d**), silver staining of supernatants (**e**), and cell viability (**f**) in BMDMs (genotypes as above) at 12 hpi. **g** Concentrations of TNF-α in supernatants from WT and *Ninj1*^-/-^ BMDMs at 16 hpi. **h** Concentrations of IL-1β in supernatants from WT, *Ninj1*^-/-^, and *Gsdmd*^-/-^ BMDMs at 16 hpi. **i** qRT‒PCR analysis of IAV-NP vRNA in WT and *Ninj1*^-/-^ BMDMs at 12 hpi (normalized to WT group). **j** Immunoblots of NINJ1 in BMDMs with/without glycine (10 mM) at 12 hpi, BS^3^-crosslinked. **k** qRT‒PCR analysis of *Ninj1* expression in BMDMs with/without glycine (10 mM) at 12 hpi (normalized to uninfected controls). **l** LDH release in WT and *Ninj1*^-/-^ BMDMs with/without glycine (10 mM) at 12 hpi. **m** Cell viability of BMDMs with/without glycine (10 mM) at 12 hpi. Data are representative of at least two independent experiments and are presented as mean ± SD. Student’s *t*-test (**f,** **i**, and **m**) or two-way ANOVA (**b**, **c**, **g**, **h**, **k**, and **l**) was applied. ns, not significant; ****p < 0.0001

To elucidate how NINJ1 affects the cell death modality and inflammatory responses during IAV infection, we compared WT and *Ninj1*^-/-^ BMDMs in several respects. Morphologically, *Ninj1*^-/-^ BMDMs exhibited a persistent balloon-like morphology, whereas WT BMDMs eventually dissociated only with their debris left (Fig. 3a). Plasma membrane leakage occurred in both (Fig. 3a, b), but PMR and the release of cellular contents, including LDH and HMGB1, were profoundly reduced by *Ninj1* knockout or mutation of one of the residues (K45Q) critical for NINJ1 oligomerization^17–19^ (Fig. 3c–e and Supplementary Fig. 2a–c). Unexpectedly, NINJ1 deficiency did not rescue BMDMs from death (Fig. 3f), which contrasts with previous observations of comorbid heat stress and infection, another PANoptosis model.^24^ Similar results were observed in THP-1 cells, although the percentage of PI^+^ cells decreased somewhat after NINJ1 ablation (Supplementary Fig. 3j–m). In pyroptosis, NINJ1 reportedly has no effect on TNF-α or IL-1β production or release,^17^ which also applies to comorbid heat stress and infection-induced PANoptosis.^24^ As expected, equal amounts of TNF-α were released (Fig. 3g). However, IL-1β release was reduced after NINJ1 ablation, and this decrease was not due to transcriptional regulation (Fig. 3h and Supplementary Fig. 2d). In comparison, such a reduction was not observed in *Gsdmd*^-/-^ BMDMs (Fig. 3h). This unexpected contrast reveals the heterogeneity of NINJ1 and GSDMD in regulating IL-1β release among different inflammatory cell death models, even if similar PANoptosis occurs. Infection with two other IAV strains yielded similar results, indicating that the impact of NINJ1 on IL-1β release is not strain specific but rather a universal phenomenon (Supplementary Fig. 2e, f). These phenotypic changes were not the result of changes in the viral load (Fig. 3d, i).

Glycine can prevent PMR by inhibiting oligomerization of NINJ1.^23^ We aimed to verify whether it also applied in IAV-induced PANoptosis. Indeed, glycine treatment prominently restrained NINJ1 oligomerization without affecting its expression (Fig. 3j, k). Like *Ninj1* knockout, glycine treatment strongly inhibited the release of LDH, HMGB1, and IL-1β, whereas cell viability was merely affected (Fig. 3l, m and Supplementary Fig. 3a–d). Glycine inhibited NINJ1 oligomerization and LDH release in a dose-dependent manner (Supplementary Fig. 3e–i) but did not have a superimposed effect on LDH release in *Ninj1*^-/-^BMDMs (Fig. 3l), which confirmed that NINJ1 is the exact target of glycine. Additionally, all of these results were similarly observed in THP-1 cells (Supplementary Fig. 3j–m) and infection with other strains of influenza (Supplementary Fig. 3e–i).

### NINJ1-mediated cell lysis is completely dependent on ZBP1 during IAV infection

**Fig. 4:**
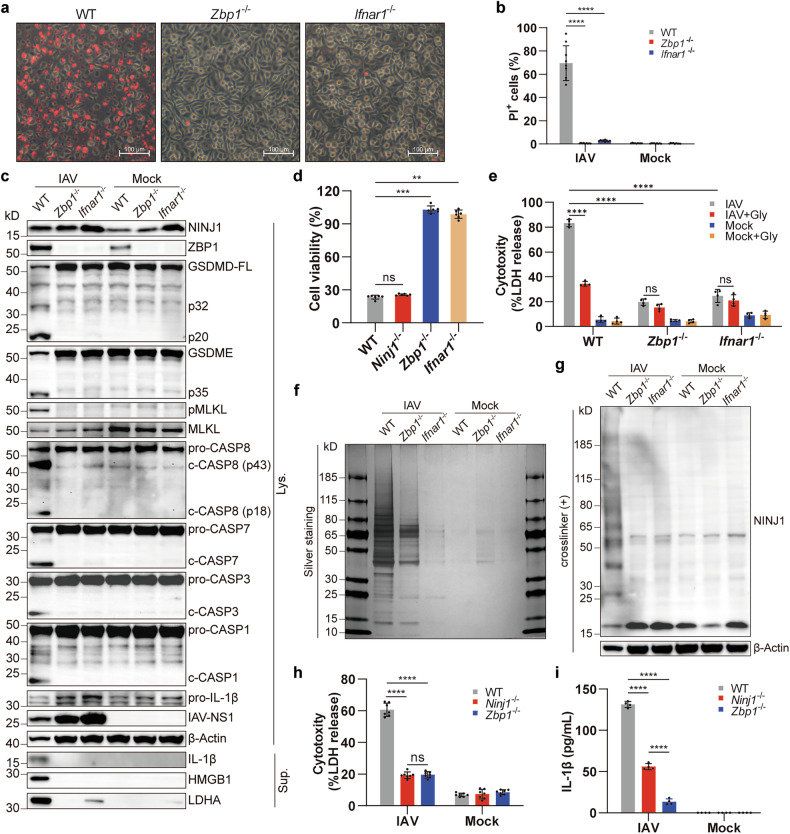
NINJ1-mediated cell lysis depends on ZBP1. **a,** **b** Representative images (**a**) and quantification (**b**) of PI^+^ cells of WT, *Zbp1*^-/-^ and *Ifnar1*^-/-^ BMDMs at 16 hpi. Scale bars, 100 μm. **c,** **g** Immunoblots of the indicated proteins in the BMDMs (genotypes as above) at 16 hpi, BS^3^-crosslinked (**g**) or non-crosslinked (**c**). **d** Cell viability of WT, *Ninj1*^-/-^, *Zbp1*^-/-^ and *Ifnar1*^-/-^ BMDMs at 16 hpi. **e** LDH release in WT, *Zbp1*^-/-^, and *Ifnar1*^-/-^ BMDMs with/without glycine (10 mM) at 16 hpi. **f** Silver staining of supernatants from (**c**). **h,** **i** LDH (**h**) and IL-1β (**i**) release in WT, *Ninj1*^-/-^, and *Zbp1*^-/-^ BMDMs at 16 hpi. Data are representative of three independent experiments and are presented as mean ± SD. Kruskal‒Wallis test (**d**) or two-way ANOVA (**b,** **e,** **h**, and **i**) was applied. ns, not significant; **p < 0.01; ***p < 0.001; and ****p < 0.0001

IAV-induced PANoptosis is type I interferon and ZBP1 dependent.^14,28^ Indeed, both PMR and cell death were reversed and all programmed cell death pathways were silenced by *Zbp1* or *Ifnar1* deficiency (Fig. 4a–f). As expected, NINJ1 oligomerization diminished simultaneously (Fig. 4g). *Zbp1*^-/-^ and *Ninj1*^-/-^ BMDMs exhibited comparable LDH release (Fig. 4h), and glycine treatment did not further reduce LDH release in *Zbp1*^-/-^ or *Ifnar1*^-/-^ BMDMs (Fig. 4e). These findings indicate that NINJ1 oligomerization and NINJ1-mediated LDH release in IAV-infected macrophages are completely dependent on ZBP1 and type I IFN responses. Although NINJ1 deficiency reduced LDH release to the same extent as ZBP1 deficiency did (Fig. 4h), *Ninj1*^-/-^ BMDMs died normally, whereas *Zbp1*^-/-^ BMDMs did not (Fig. 4d). The inhibition of IL-1β release was more thorough in *Zbp1*^-/-^ BMDMs than in *Ninj1*^-/-^ BMDMs (Fig. 4i). Together, our results indicate that NINJ1 oligomerization and cell lysis mediated by it are dependent on ZBP1 in IAV-induced PANoptosis and that NINJ1 differs from ZBP1 in its role in cell viability and inflammatory responses.

### NINJ1 oligomerization is not regulated by the NLRP3 inflammasome

Numerous studies have explored the essential role of the NLRP3 inflammasome in both innate and adaptive immunity during IAV infection.^11–13^ We found that knocking out NLRP3 inflammasome components did not abrogate IAV-induced lytic cell death (Supplementary Fig. 4a–d), similar to previous studies.^28^ Accordingly, NINJ1 oligomerization was still retained (Supplementary Fig. 4e), and PANoptosis pathways remained intact, including GSDMD activation (Supplementary Fig. 4f). Additionally, IL-1β release was only partially reduced by NLRP3 inflammasome deficiency (Supplementary Fig. 4f). This is not contradictory since GSDMD and pro-IL-1β can be cleaved by Caspase-8.^29–31^ Overall, the NLRP3 inflammasome does not participate much in IAV-induced macrophage PANoptosis or NINJ1 oligomerization except for a minor role in IL-1β maturation.

### Activation of any cell death pathway in IAV-induced PANoptosis is sufficient to induce NINJ1 oligomerization

**Fig. 5:**
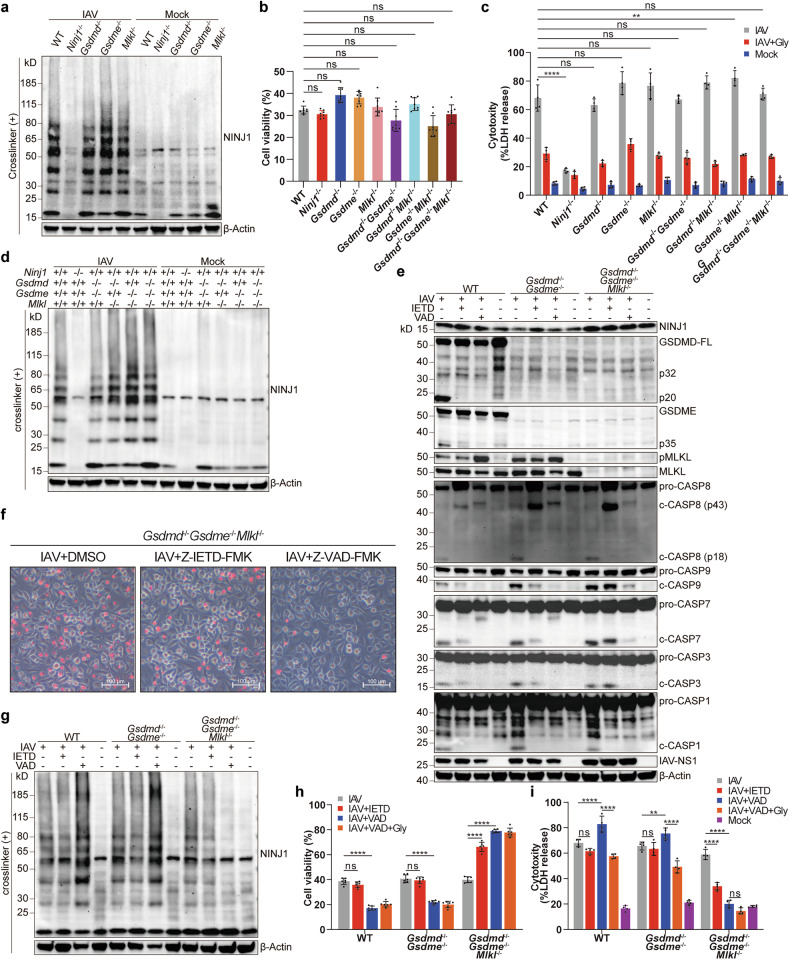
Activation of any PANoptosis pathway induces NINJ1 oligomerization during IAV infection. **a,** **d** Immunoblots of NINJ1 in BMDMs of indicated genotypes at 16 hpi, BS^3^-crosslinked. **b** Viability of BMDMs of indicated genotypes at 16 hpi. **c** LDH release in BMDMs of indicated genotypes at 16 hpi, with/without glycine (10 mM). **e,** **g** Immunoblots of indicated proteins in WT, *Gsdmd*^-/-^*Gsdme*^-/-^, and *Gsdmd*^-/-^*Gsdme*^-/-^*Mlkl*^-/-^ BMDMs at 16 hpi, treated with DMSO, Z-IETD-FMK (25 μM), or Z-VAD-FMK (25 μM), BS^3^-crosslinked (**g**) or non-crosslinked (**e**). **f** Representative images of PI^+^ cells from panel (**e**). Scale bars, 100 μm. See also Supplementary Fig. 5f. **h**, **i** Cell viability (**h**) and LDH release (**i**) in WT, *Gsdmd*^-/-^*Gsdme*^-/-^, and *Gsdmd*^-/-^*Gsdme*^-/-^*Mlkl*^-/-^ BMDMs at 16 hpi, treated with DMSO, Z-IETD-FMK, Z-VAD-FMK, or Z-VAD-FMK plus glycine. Data are representative of three independent experiments and are presented as mean ± SD. Kruskal‒Wallis test (**b**) or two-way ANOVA (**c**, **h**, and **i**) was applied. ns, not significant; **p < 0.01; and ****p < 0.0001

Considering that GSDMD cleavage, GSDME cleavage, and MLKL phosphorylation, together with NINJ1 oligomerization, were all abrogated in *Zbp1*^-/-^ BMDMs (Fig. 4c, g), we wondered whether there is a certain association between these pore-forming executioners and NINJ1. We first knocked out each of these pore-forming proteins. NINJ1 oligomerization and lytic cell death were not affected at all (Fig. 5a–c and Supplementary Fig. 5a–c). This isn’t beyond expectation since functional redundancy is quite common among cell death pathways. We then proposed that deficiency of all lytic cell death pathways, namely, pyroptosis and necroptosis, would lead to failure of NINJ1 oligomerization. Surprisingly, not only *Gsdmd*^-/-^*Gsdme*^-/-^, *Gsdmd*^-/-^*Mlkl*^-/-^, and *Gsdme*^-/-^*Mlkl*^-/-^ BMDMs but also *Gsdmd*^-/-^*Gsdme*^-/-^*Mlkl*^-/-^ BMDMs underwent robust cell lysis, reaching a level similar to that of their WT counterparts (Fig. 5b, c and Supplementary Fig. 5a, b, d, e). Comparable NINJ1 oligomerization was observed in the macrophages of all these genotypes (Fig. 5d). The inhibition of LDH release by glycine treatment (Fig. 5c) further confirmed that NINJ1 oligomerized normally. Only the apoptosis pathway was intact in *Gsdmd*^-/-^*Gsdme*^-/-^*Mlkl*^-/-^ BMDMs (Fig. 5e), which is conventionally thought to be immunologically silent. However, we failed to convert the inflammatory cell death to a relatively immunologically silent one, namely, apoptosis in a traditional sense.

Then what is the trigger of NINJ1 oligomerization in the absence of the pyroptosis and necroptosis pathway? Considering that the apoptosis pathway is the only activated PANoptosis pathway underlying the difference between *Zbp1*^-/-^ and *Gsdmd*^-/-^*Gsdme*^-/-^*Mlkl*^-/-^ BMDMs, one possibility is that the apoptosis pathway alone is sufficient to activate NINJ1 to induce cell lysis. The other is that NINJ1 functions independently of the pyroptosis, necroptosis and apoptosis pathways. Therefore, we treated *Gsdmd*^-/-^*Gsdme*^-/-^*Mlkl*^-/-^ BMDMs with the pan-caspase inhibitor Z-VAD-FMK to inhibit all apoptosis pathways. Since GSDMD and GSDME are already deficient, there is no need to consider the effects of Z-VAD-FMK’s effect on pyroptosis-inducing caspases. After treatment, all programmed cell death pathways were blocked (Fig. 5e), followed by decreased NINJ1 oligomerization and lytic cell death (Fig. 5f–i and Supplementary Fig. 5f–h). Moreover, supplementation with glycine did not further reduce LDH release (Fig. 5i). Therefore, activation of the apoptosis pathway could induce NINJ1 oligomerization to rupture the plasma membrane, even in the absence of the pyroptosis and necroptosis pathway. In comparison, NINJ1 oligomerization and LDH release were not decreased but were even increased in WT BMDMs after Z-VAD-FMK treatment (Fig. 5g, i). So were in *Gsdmd*^-/-^*Gsdme*^-/-^ BMDMs, whose MLKL was phosphorylated as usual (Fig. 5e, g, i). Glycine treatment also partially inhibited NINJ1 oligomerization and LDH release in Z-VAD-FMK-treated *Gsdmd*^-/-^*Gsdme*^-/-^ BMDMs (Fig. 5i and Supplementary Fig. 5i). NINJ1 was formerly reported to function little in necroptosis.^17,22,23^ However, here we showed that in the absence of the apoptosis and pyroptosis pathways, necroptosis could also induce NINJ1 oligomerization, and inhibiting NINJ1 oligomerization could partially alleviate necroptosis-induced cell lysis during IAV infection. Treatment with the caspase-8 inhibitor Z-IETD-FMK yielded results showing similar trends, though NINJ1 oligomerization and LDH release were only partially inhibited (Fig. 5e–i and Supplementary Fig. 5f–h). The remaining apoptosis pathway was not thoroughly inhibited, as the cleavage of Caspase-3 and Caspase-7, which are possibly cleaved by Caspase-9, could still be detected. The activation of Caspase-9 disappeared when we substituted Z-IETD-FMK with Z-VAD-FMK (Fig. 5e), which inhibited NINJ1 oligomerization more thoroughly (Fig. 5g). Together, our results suggest that NINJ1 responds to the activation of any of the three classical cell death pathways of PANoptosis and that even the apoptosis pathway alone is able to induce robust cell lysis by inducing NINJ1 oligomerization (Supplementary Fig. 6).

### NINJ1 functions poorly in IAV-infected alveolar epithelial cells

While the expression of NINJ1 in AECs was much lower than that in myeloid cells (Fig. 1f), its potential role in AECs could not be ruled out since NINJ1 was also upregulated in AECs (Fig. 1g and Supplementary Fig. 7b). In contrast to that in macrophages, GSDME-mediated pyroptosis is the major mode of cell death in IAV-infected AECs.^32–34^ Therefore, we compared the death patterns of IAV-infected WT, *Ninj1*^-/-^ and *Gsdme*^-/-^ A549 cells. Improved cell viability, attenuated cell lysis, and decreased leakage of intracellular components were observed only in *Gsdme*^-/-^ A549 cells but not in *Ninj1*^-/-^ A549 cells (Supplementary Fig. 7a–d). Accordingly, glycine treatment had no effect on LDH release (Supplementary Fig. 7e). Since respiratory epithelial cells provide niches for IAV replication and the production of progeny virions, we determined the viral titers in the culture supernatants and found no significant difference between WT and *Ninj1*^-/-^ A549 cells (Supplementary Fig. 7f). Although direct evidence of NINJ1 oligomerization in A549 cells is temporarily unavailable because of a lack of appropriate detection antibodies, we may conclude that NINJ1 plays little role in IAV-infected AECs.

### NINJ1 drives IAV-induced lung pathology and excessive inflammation

**Fig. 6:**
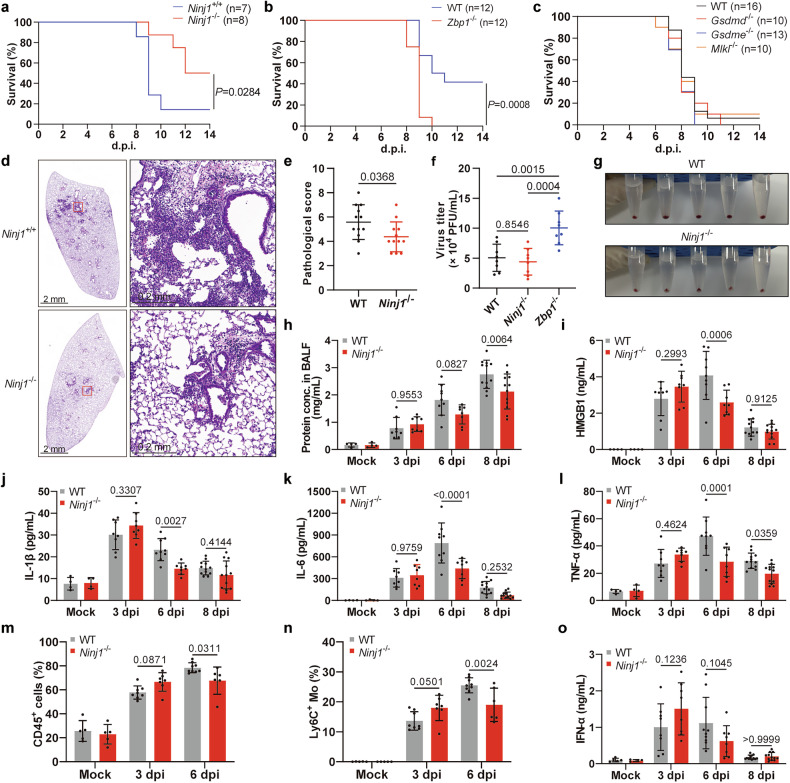
NINJ1 drives IAV-induced lung pathology and hyperinflammation. **a**−**c** Survival curves of (**a**) *Ninj1*^-/-^ mice and their littermates; (**b**) *Zbp1*^-/-^ and WT mice; (**c**) *Gsdmd*^-/-^, *Gsdme*^-/-^, *Mlkl*^-/-^, and WT mice. **d,** **e** H&E staining (**d**) and pathological scores (**e**) of the same lung lobe of WT and *Ninj1*^-/-^ mice at 5 dpi. The right panel in (**d**) shows magnified images of the insects. Scale bars, 2 mm and 0.2 mm (insects). **f** Viral titers in lungs of WT, *Ninj1*^-/-^ and *Zbp1*^-/-^ mice at 5 dpi. **g** Cellular pellets in BALF of WT and *Ninj1*^-/-^ mice at 8 dpi after centrifugation. **h**−**l**, **o** Concentrations of total proteins (**h**) and indicated cytokines (**i**−**l**, **o**) in BALF of WT and *Ninj1*^-/-^ mice at 3, 6, and 8 dpi. **m**, **n** Frequencies of CD45^+^ cells (**m**) and Ly6C^+^ monocytes (**n**) among total live cells in BALF of WT and *Ninj1*^-/-^ mice at 3 and 6 dpi. Gating strategies are shown in Supplementary Fig. 9a. Mice were infected with LD_100_ (**a,** **c**) or LD_50_ (**b,** **d**–**o**) of IAV. Data are representative of at least two independent experiments and are presented as mean ± SD. Two-way ANOVA (**h**‒**o**), one-way ANOVA (**f**), or Student’s *t*-test (**e**) was used. Survival curves were analyzed by the log-rank test (**a**−**c**)

Several in vivo studies have revealed the potential role of NINJ1 in different diseases, but no studies have investigated viral pneumonia. Unlike *Zbp1* deficiency, *Ninj1* deficiency improved survival after IAV challenge (Fig. 6a, b). In line with the in vitro results, *Gsdmd*^-/-^, *Gsdme*^-/-^, and *Mlkl*^-/-^ mice did not benefit from gene deficiency (Fig. 6c). Pathological injuries were apparently alleviated in *Ninj1*^-/-^ mice (Fig. 6d, e). The protection conferred by *Ninj1* deficiency was not a result of changes in the viral load (Fig. 6f). Compared with that from WT mice, the BALF (bronchoalveolar lavage fluid) from *Ninj1*^-/-^ mice contained fewer cellular pellets after centrifugation and lower protein concentrations, and significantly lower levels of HMGB1 and proinflammatory cytokines (IL-1β, IL-6, and TNF-α) were detected (Fig. 6g–l). These differences were most pronounced during the severe infection phase (6 or 8 dpi) rather than during the early infection phase (3 dpi), although the exact time points of significant contrast differed slightly between individual markers. In contrast, more proteins and HMGB1, as well as comparable IL-1β and TNF-α levels, were observed in the BALF from *Gsdmd*^-/-^ mice (Supplementary Fig. 8a–d). *Zbp1*^-/-^ mice bore a greater viral burden and more proteins in the BALF (Fig. 6f and Supplementary Fig. 8a), possibly due to failure to eradicate IAV infection. Although excessive IL-1β mediates the immunopathology of hyperinflammation,^35,36^ IL-1β is fundamental to host defense against flu challenge.^12,37^ Both *Il1r*^-/-^ and *Il1b*^-/-^ mice were more susceptible to IAV-induced mortality (Supplementary Fig. 8e, f), and additional ablation of *Il1b* weakened the protection conferred by *Ninj1* deficiency (Supplementary Fig. 8g). Therefore, it seems that NINJ1 decreases IL-1β release to an extent that does not sacrifice its anti-viral immunity. Since inflammatory cell infiltration was reduced (Fig. 6d), to better understand the immunological changes caused by *Ninj1* knockout, we analyzed BALF cells via flow cytometry. The immune cell subset composition showed no difference at 3 dpi (Fig. 6m, n and Supplementary Fig. 8h, 9a–c), ensuring normal inflammatory responses to clear the virus. Reduced immune infiltration primarily occurred at 6 dpi, specifically affecting Ly6C^+^ monocytes (Fig. 6m, n and Supplementary Fig. 8h), which are closely linked to IAV-induced lung injury.^38,39^ Significant differences were not detected in the type I interferon responses (Fig. 6o) or anti-IAV CD8^+^ T cell responses (Supplementary Fig. 8i, 9c). The impaired epithelial barrier integrity was ameliorated after *Ninj1* knockout, as shown by less reduced expression of the tight junction protein ZO-1 (Supplementary Fig. 8j). This is likely an indirect consequence of *Ninj1* knockout (reduced immune inflammation) rather than a direct result, since in vitro experiments (Supplementary Fig. 7) did not provide direct evidence that NINJ1 plays a significant role in AECs. Notably, we excluded *Ninj1*^-/-^ and *Ninj1*^-/-^*Il1b*^-/-^ mice from studies that developed abnormally in appearance, i.e., stunted growth, hydrocephaly, and ataxia (Supplementary Fig. 8k), as reported in the Mouse Genome Informatics (MGI) database.^40^

### NINJ1 is a potential bioindicator of hyperinflammation and poor outcome in patients with severe viral pneumonia

Although we revealed a potential association between elevated NINJ1 and disease severity in IAV-infected murine lungs (Fig. 1), whether this relationship also applies in the clinic remains unknown. Since BAL is not a routine procedure for influenza unless clinically indicated, BALF samples from severe IAV-infected patients were temporarily unavailable to us in the short term, and we did not find appropriate scRNA-seq data on clinical respiratory samples in public databases. We noted that PANoptosis is involved not only in IAV infection but also in SARS-CoV-2 infection.^41–44^ Like IAV, SARS-CoV-2 is a single-stranded RNA virus that causes severe pneumonia as well as mild upper respiratory infection. Since influenza and COVID-19 share many similarities in terms of virology and pathophysiology, we sought to verify the role of NINJ1 in COVID-19 patients. We analyzed scRNA-seq data from BALF samples collected by our group from 10 hospitalized COVID-19 patients and 5 controls (CNCB, PRJCA033941; Supplementary Table 2). All 10 patients were severely/critically ill, among whom 5 died eventually and 5 survived. We examined NINJ1 expression globally in the three groups and found that NINJ1 was profoundly elevated after infection and was most abundantly expressed in the deceased group (Supplementary Fig. 10a). The cells were clustered into 13 major types (Supplementary Fig. 10b, c). The proportions of monocytes/macrophages (Mo/Mas) and Neus increased, whereas the proportion of alveolar macrophages (AMs) decreased after SARS-CoV-2 infection (Supplementary Fig. 10d). This change in cell fraction was more significant in patients who died, indicating that Mo/Mas and Neus might play important roles in COVID-19 disease progression. We further assessed NINJ1 expression patterns in different cell types and found that NINJ1 was highly expressed specifically in Mo/Ma and Neu and exhibited a progressive increase among the three groups (Supplementary Fig. 10e). None of the other PANoptosis-associated genes exhibited trends similar to those of NINJ1 in Mo/Mas (Supplementary Fig. 10e). The expression of NINJ1 was positively correlated with inflammatory responses but not with antiviral responses both globally and in Mo/Mas, with a correlation coefficient that was not much lower than that of IL1B (Supplementary Fig. 10f–h). Although NINJ1 was also highly expressed and showed a stepwise increase in Neus, a positive correlation was not detected in Neus or AMs (Supplementary Fig. 10f). Owing to the limited sample size of our study, we re-analyzed another single-nuclei RNA sequencing data from a published dataset (GSE171524^45^) comprising data directly from lung specimens from COVID-19 patients within hours of death. These samples had advantages over our samples since lung specimens could provide a more comprehensive view of SARS-CoV-2-induced immunopathology than BALF samples. After cell clustering (Supplementary Fig. 11a, b), we found that NINJ1 was specifically highly expressed similarly in increased Mo/Mas (Supplementary Fig. 11c, d). In line with our results, the expression of NINJ1 was aberrantly increased in Mo/Mas from deceased COVID-19 patients (Supplementary Fig. 11e). Collectively, our scRNA-seq analysis reveals the potential of NINJ1 in Mo/Mas to be a biomarker of hyperinflammation status and poor prognosis in patients with severe COVID-19. Further analysis of other viral pneumonia based on a larger sample size is warranted.

## Discussion

Although NINJ1 was first reported as a mediator of PMR 4 years ago,^17^ little effort had been made to explore NINJ1’s role in viral infection prior to this study. Our study provides insight into the correlation between NINJ1 and the disease progression of influenza and COVID-19, as well as the mechanism underlying how NINJ1 functions during IAV-induced PANoptosis. Specifically, different doses of IAV led to a dose-dependent increase in NINJ1 in myeloid cells in vivo. Mechanistically, NINJ1 mediates cell lysis, DAMP release, and partial IL-1β release during IAV-induced PANoptosis without affecting the progression of cell death. The activation of any of the three pathways involved in PANoptosis, including the apoptosis pathway, induced NINJ1 oligomerization to a similar extent. Mortality and immunopathology were partially alleviated in *Ninj1*^-/-^ mice. Furthermore, we extend our conclusion to COVID-19 and reveal an association of NINJ1 expression in monocytes and macrophages with disease prognosis and inflammatory responses.

The most concerning question about NINJ1 is the exact trigger and the underpinnings of NINJ1 oligomerization. Different structural biological models have been proposed to explain the structural basis of NINJ1-mediated PMR,^18–21^ but how NINJ1 is activated remains unknown. Additionally, it is unclear whether other cell death models share the same trigger as pyroptosis does. Indeed, in IAV-induced PANoptosis, we reproduced and verified most NINJ1-related phenotypes observed in pyroptosis, including inhibited cell lysis and DAMP release, as well as unaltered cell viability and the release of most cytokines. A key distinction lies in the opposing roles of GSDMD and NINJ1 in IL-1β release. While GSDMD knockout has no impact, NINJ1 knockout significantly inhibits IL-1β release—a reversal of the canonical pyroptosis mechanism. One possible explanation is that active fragments of GSDMD (p32) can be cleaved into an inactive form (p20) by Caspase-3/7^46^ (Fig. 3d), resulting in impaired regulation of IL-1β release. Inhibition of this dual cleavage of GSDMD enhances IL-1β release.^47^ We are not the first to explore the role of NINJ1 in PANoptosis. Han et al. reported that NINJ1 is a mediator of PANoptosis during infection conditions and heat stress.^24^ In that PANoptosis model, NINJ1 deficiency surprisingly rescued BMDMs from death.^24^ Therefore, the roles of NINJ1 are not always the same among different types of cell death, especially when more cell death pathways are involved. After all, even PANoptosis models vary from each other.

Viral sepsis due to aberrant cytokine release and unchecked cell death are the important pathophysiology of severe viral pneumonia. Converting an inflammatory cell death to an immunologically silent one without sacrificing viral clearance is a popular guiding ideology, but our unsuccessful attempt to switch PANoptosis to apoptosis in *Gsdmd*^-/-^*Gsdme*^-/-^*Mlkl*^-/-^ BMDMs (Fig. 5) has raised questions about this hypothesis. Only when the apoptosis pathway is blocked together or when NINJ1 is ablated individually can cell lysis be terminated. This means that during IAV infection, activation of the apoptosis pathway is inflammatory and not equal to apoptosis in the traditional sense due to the activation of NINJ1. Clearly, this macrophage inflammatory death mediated solely by a single apoptotic pathway is not the so-called secondary necrosis after apoptosis, because the latter is mediated by GSDME,^48^ which we have already knocked out.

The ability to prevent PMR without compromising cell death or necessary inflammatory responses makes NINJ1 a potential therapeutic target in IAV infection, which was verified in in vivo studies. *Ninj1*^-/-^ mice exhibited improved survival, alleviated pathology, and decreased inflammatory responses. The inflammatory response to infection is a double-edged sword: insufficient inflammation fails to clear pathogens, whereas excessive inflammation causes immunopathology. For example, the roles of the NLRP3 inflammasome and IL-1β in influenza infection remain debated. Studies on genetically deficient mice, including ours (Supplementary Fig. 8e–g), generally show reduced survival,^11–13,37^ but some inhibitor-based studies have yielded opposite results.^36,49,50^ These findings suggest that controlling inflammation during disease progression may be more beneficial than suppressing it from the outset. Coincidentally, early inflammatory responses were not affected by *Ninj1* deficiency (Fig. 6h–o and Supplementary Fig. 8h), which guaranteed necessary anti-IAV immunity. The in vitro results revealed that *Ninj1* deficiency only partially inhibited IL-1β release, unlike the near-complete suppression observed with *Zbp1* knockout (Fig. 4i). Additionally, in vivo data revealed that the IL-1β reduction is optimal: ablating *Il1b* in *Ninj1*^-/-^ mice abolishes the protective phenotype conferred by *Ninj1* knockout (Supplementary Fig. 8g).

We also reexamined the phenotypes of several genetically modified mice. We did not observe a survival improvement in *Gsdmd*^-/-^, *Mlkl*^-/-^, or *Zbp1*^-/-^ mice as previously reported.^7–10,28^ This nonconformity has also been mentioned by other studies,^14,30^ indicating that these protective phenotypes are not robust enough to counteract the variations in viral doses and strains.

Although all abnormally developed *Ninj1*^-/-^ mice were excluded, as mentioned previously (Supplementary Fig. 8k), we cannot rule out other potential abnormities, which do not manifest in appearance but might directly or indirectly affect susceptibility to infection. Thus, macrophage-selective knockout of *Ninj1* or specific inhibitor treatment may be a better choice. However, treatment targeting NINJ1 is still unavailable except for an anti-NINJ1 antagonist antibody developed by Kayagaki et al. ^51^, which limits its in vivo application.

Through scRNA-seq analysis, we associated the expression level of NINJ1 in macrophages with the severity and prognosis of influenza and COVID-19. Existing studies establish that NINJ1 functionality primarily depends on its oligomerization; however, the relationship between NINJ1 expression levels and PMR remained unexplored. During the revision of this manuscript, Zhu et al. published pivotal work demonstrating that NINJ1 regulates plasma membrane fragility under mechanical strain, with NINJ1 levels inversely correlating with the force required for PMR.^52^ Thus, although the precise mechanisms driving NINJ1 upregulation upon IAV or SARS-CoV-2 infection remain unclear, this elevation may represent a cellular preparatory step to facilitate membrane disintegration. Their findings provide a mechanistic framework for the correlation we identified between NINJ1 expression and disease severity. However, we admit that this conclusion is not robust and should be treated with caution because of the lack of data on influenza patients and the limited sample size of COVID-19 patients. Further verification on data from a larger sample size as well as a wider range of viral pneumonia cases is warranted. Additionally, unfortunately, we cannot reproduce our in vitro findings in SARS-CoV-2 infection due to no access to facilities with a relatively high biosafety level (BSL-3 or BSL-4). It is worth mentioning that neutrophils are actually the cell type where *Ninj1* is most highly expressed. The expression patterns of NINJ1 in both IAV- and SARS-CoV-2-infected neutrophils were similar. The correlation between the NINJ1 expression level and inflammation in total cells and macrophages was not detected in neutrophils (Supplementary Fig. 10f), suggesting a different but possibly important role of NINJ1 in neutrophils. To date, few efforts have been made to explore the role of NINJ1 in neutrophils, which will be addressed in our next study.

In summary, our study offers insights into the role of NINJ1 in immunopathology during IAV infection and reveals its potential as a therapeutic target and bioindicator in viral pneumonia and viral sepsis. Further studies exploring NINJ1 in other viral infections and in other cell types are warranted.

## Materials and methods

### In vitro IAV infection

BMDMs were seeded into 12-well plates at a density of 8 × 10^5^ cells/well and infected with the indicated IAV strain at an MOI of 20 in DMEM. iBMDMs were seeded into 12-well plates at a density of 2 × 10^6^ cells/well and infected with PR8 at an MOI of 20 in DMEM. THP-1 cells were seeded into 12-well plates in 10% FBS-containing DMEM at a density of 5 × 10^5^ cells/well and differentiated into macrophages with 100 ng/ml phorbol 12-myristate 13-acetate (PMA; Sigma Aldrich, P1585) for 2 days. Then, fresh media without PMA took the place for 12 h. Differentiated THP-1 cells were infected with PR8 at an MOI of 20 in DMEM. A549 cells were infected with PR8 at an MOI of 1 in Opti-MEM™ I Reduced Serum Medium. After absorption for 2 h, the cells were washed with PBS three times and then maintained in fresh Opti-MEM™ I Reduced Serum Medium (Gibco, 11058021) for the indicated times. All in vitro infection studies were performed in a BSL-2 laboratory.

### Immunoblot analysis

The cells were lysed in situ with RIPA buffer containing protease and phosphatase inhibitors (Roche, 11697498001 and 4906845001). Proteins in supernatants were enriched by trichloroacetic acid/acetone precipitation and resuspended in ddH_2_O. The collected proteins were denatured by NuPAGE™ LDS Sample Buffer (Invitrogen, NP0008) supplemented with NuPAGE™ Sample Reducing Agent (Invitrogen, NP0009) and boiled at 70°C for 10 min. Denatured proteins were separated via 4 − 20% Bis-Tris gel (ACE Biotechnology, ET12420Gel) electrophoresis in MOPS buffer (ACE Biotechnology, BR0001-02) and transferred to PVDF membranes (Millipore, ISEQ00010 and IPVH00010). The blots were blocked with 5% skim milk, followed by incubation with the indicated primary antibodies (1:1000) at 4°C overnight, and then secondary antibodies (1:5000) for 1 h at room temperature. The blots were developed via enhanced chemiluminescence (ECL) (Millipore, WBULS0500) on a ChemiDoc™ MP Imaging System (Bio-Rad, 734BR5056). The antibodies used for immunoblotting were listed in Supplementary Table 3.

### Crosslinking assays

To detect NINJ1 oligomerization, the cells were incubated in Hanks’ balanced salt solution (Gibco, 14025092) containing 2.5 mM BS^3^ crosslinker (Thermo Scientific, A39266) for 10 min. The crosslinking reaction was quenched by adding 1 M Tris-HCl, pH 7.5, to reach a final concentration of 50 mM. The cell lysates were then collected as described above.

### Silver staining of total proteins in culture supernatants

After the denatured proteins in the supernatants were separated via 4 − 20% Bis-Tris gel electrophoresis, the gels were silver stained via a Pierce™ Silver Stain Kit (Thermo Scientific, 24612) and imaged on a ChemiDoc™ MP Imaging System.

### Generation of *Ninj1*^-/-^ iBMDMs, THP-1 cells, and A549 cells

CRISPR-Cas9-mediated ablation of the *Ninj1* gene in *Ninj1*^-/-^ iBMDMs, THP-1 cells, and A549 cells was achieved with CRISPR-Cas9 RNPs. In brief, the Cas9 protein (New England Biolabs, M0646 or Ubigene, YK-Cas9-50) and gRNA were incubated at room temperature for 10–20 min, followed by electroporation via a Neon transfection system (Thermo Scientific, MPK5000) according to the manufacturer’s instructions. After 2 days, the cells were sorted into 96-well plates via a BD FACSAria™ III cell sorter to form single clones. Candidate clones were screened via genomic sequencing to verify the presence of mutations in both alleles. The sequences of the gRNAs used were listed in Supplementary Table 4. *Ninj1*^-/-^ iBMDMs were constructed with the help of Haixing Bioscience (Suzhou, China), whereas *Ninj1*^-/-^ A549 cells and THP-1 cells were constructed with the help of Ubigene Biosciences (Guangzhou, China).

### Stable expression of NINJ1^WT^ and NINJ1^K45Q^ in *Ninj1*^-/-^ iBMDMs

Packaging, production, and titration of lentiviruses expressing NINJ1^WT^ or NINJ1^K45Q^ were accomplished by Genomeditech (Shanghai, China). *Ninj1*^-/-^ iBMDMs were transduced with lentivirus and then selected with puromycin at a concentration of 2.5 μg/mL.

### Cytotoxicity assay

IAV-induced cytotoxicity was determined by LDH release in the cell culture supernatants via the CytoTox 96^®^ Non-Radioactive Cytotoxicity Assay Kit (Promega, G1780) according to the manufacturer’s instructions.

### Measurement of cytokine levels

IL-1β (Invitrogen, 88-7013a-88; ABclonal, RK04878), HMGB1 (Elabscience, E-EL-M0676), TNF-α (Invitrogen, 88-7324-88), and IL-6 (Invitrogen, 88-7064-88) levels in cell culture supernatants or murine BALF were determined via ELISA kits according to the manufacturer’s instructions.

### Cell viability assay

Cell viability after IAV infection was determined via the CellTiter-Glo^®^ Luminescent Cell Viability Assay Kit (Promega, G7571) according to the manufacturer’s instructions.

### qRT‒PCR

The RNA of the cell and tissue samples was extracted with TRIzol reagent (Invitrogen, 15596018CN) according to the manufacturer’s instructions. The RevertAid First Strand cDNA Synthesis Kit (Thermo Scientific, K1622) was used for reverse transcription, and the PowerUp SYBR Green Master Mix (Applied Biosystems, A25742) was used for real-time fluorescent quantitative PCR detection. Real-time qPCR was performed on a QuantStudio™ 5 Real-Time PCR System (Applied Biosystems, A28569). The sequences of the primers used for qRT‒PCR were listed in Supplementary Table 5. The fold changes were calculated with *Gapdh* as the internal reference. The results were normalized to those of the WT group.

### Animal IAV infection study

Age- and sex-matched 8- to 10 week-old mice were challenged intranasally with the indicated dose of PR8 (LD_100_/male: 1 × 10^4^ PFU; LD_100_/female: 2 × 10^3^ PFU; LD_50_/male: 4 × 10^3^ PFU; LD_50_/female: 1 × 10^3^ PFU) after being anesthetized with Avertin. We observed the mice for 14 days to plot survival curves. For *Ninj1*^-/-^ mice, littermate *Ninj1*^+/+^ mice served as controls. For other mice, co-housing wild-type mice on the same background served as a control. *Ninj1*^-/-^ and *Ninj1*^-/-^*Il1b*^-/-^ mice exhibiting stunted growth, hydrocephaly, and ataxia were excluded from any experiment in this study. Lungs were harvested at the indicated days post infection. For histological and immunohistochemical analysis, lungs were fixed in 4% paraformaldehyde overnight and then prepared into paraffin sections for H&E staining and immunofluorescence staining. To determine the viral titers in the lungs, the lungs were homogenized in 1 ml of PBS, and the supernatants were collected after centrifugation. The viral titers were subsequently determined via plaque assays on MDCK cells. BALF samples were collected in 1 ml of PBS. After centrifugation, the supernatants were used for determining the concentrations of cytokines via ELISA, while the cell pellets were used for flow cytometric analyses. The total protein concentration in the BALF was determined via a Pierce™ BCA Protein Assay Kit (Thermo Scientific, 23227).

### Flow cytometric analyses of cells in murine BALF and lungs

Lung tissues were cut into pieces and digested into single cells by collagenase I. BALF cells were collected directly from the cell pellets after centrifugation. Red blood cells were lysed via RBC lysis buffer (Invitrogen, 00-4333-57). After being stained with fixable viability dyes (Zombie UV™ dye [BioLegend, 423108] or LIVE/DEAD fixable dye [Invitrogen, L34982]) and blocked with TruStain FcX™ (anti-mouse CD16/32) Antibody (BioLegend, 101320), single cells were stained with different combinations of the antibodies listed in Supplementary Table 6 and T-Select H-2D^b^ Influenza NP Tetramer-ASNENMETM-PE (MBL, TS-M508-1). Gating strategies were shown in Supplementary Fig. 9. The samples were analyzed on a CytoFLEX LX flow cytometer (Beckman, C06779).

### Statistical analysis

Statistical analysis was performed via GraphPad Prism v9.5.1. The data presented in this study are representative of at least two independent experiments. The data are presented as the mean ± SD. For comparisons between two groups, Student’s *t-*test was used to determine the statistical significance. For three or more groups, one-way ANOVA (data normally distributed) or the Kruskal‒Wallis test (data not all normally distributed) was applied if there was only one independent variable, and two-way ANOVA was applied if there were two independent variables. The log-rank test was used to compare the survival curves. *p* < 0.05 was considered statistically significant (*p < 0.05, **p < 0.01, ***p < 0.001, and ****p < 0.0001).

## Supplementary information

Supplementary Materials

Table S1. Meta information of IAV-infected murine lungs

Table S2. Demographics and clinical characteristics of 15 human samples

## Data Availability

The RNA-seq and scRNA-seq data from our own group have been deposited at the China National Center for Bioinformation (CNCB, https://www.cncb.ac.cn/) under accession numbers PRJCA034048, PRJCA034049, and PRJCA033941, which will be publicly available upon the publication of this article. The publicly available single-nuclei RNA sequencing data of lung specimens from COVID-19 patients are available in the GEO database under accession number GSE171524. Our analysis code for the scRNA-seq data has been uploaded to GitHub (https://github.com/Yitian-Xu/scRNA_of_NINJ1). Any additional information required to re-analyze the data reported in this paper should be directed to Bin Cao (caobin_ben@163.com) and is available upon request.
