# Supplementary material for: Ninjurin-1 mediates cell lysis and detrimental inflammation of PANoptosis during influenza A virus infection
Source: Signal Transduct Target Ther. 2025 Sep 23;10:307. doi: 10.1038/s41392-025-02391-9 (PMC12454662; doi:10.1038/s41392-025-02391-9)
Supplement: Supplementary file 1 — Supplementary Materials [file 41392_2025_2391_MOESM1_ESM.docx]

Supplementary Materials for

Ninjurin-1 mediates cell lysis and detrimental inflammation of PANoptosis during influenza A virus infection

Yitian Xu,^1,2,#^ Ying Zheng,^2,3,#^ Yan Liu,^4,#^ Cheng Wei,^2,#^ Juan Ren,^2,5^ Wenting Zuo,^1,2^ Runqing Gu,^6^ Hongyu Liu,^2,3^ Xiaoyan Deng,^7^ Yaxue Liu,^8^ Xiao Shang,^8^ Weiwei Ge,^7^ Ziyao Li,^2^ Yijiao Huang,^8^ Di He,^2,3^ Xuhui Shen,^2^ Zeyi Wang,^2^ Chen Lyu,^1,2^ Zai Wang,^2^ Yuxian Mu,^1,2^ Zihuan Zhang,^1^ Hongping Wu,^2,*^ Haibo Li,^2,*^ Bin Cao^1,2,3,5,7,*^

Correspondence to: caobin_ben@163.com

**This PDF file includes:**

Supplementary Materials and Methods

Figures S1 to S11

Tables S3 to S6

References in Supplementary Materials

**Other Supplementary Materials for this manuscript include the following:**

Tables S1 to S2

**Supplementary Materials and Methods**

**Isolation and culture of BMDMs**

Primary BMDMs were isolated from 8-week-old male mice according to the protocols of Toda *et al*.^1^ The isolated BMDMs were cultivated for differentiation for 6 days in IMDM (Gibco, C12440500BT) supplemented with 10% FBS, 30% L929-conditioned media, 1X MEM non-essential amino acids (Gibco, 11140050), 100 U/mL penicillin and 100 μg/mL streptomycin (Gibco, 15140122). Mature BMDMs were then seeded into 12-well plates at a density of 8×10^5^ cells/well for subsequent IAV infection.

**IAV propagation and titration**

Influenza A virus A/Puerto Rico/8/34 (H1N1) (ATCC, VR-95), A/WS/33 (H1N1) (ATCC, VR-1520), and A/Wisconsin/67/2005 (H3N2) (ATCC, VR-1881) were propagated in the allantoic cavity of 9- to 11-day-old embryonated chicken eggs (Beijing Boehringer Ingelheim Vital Biotechnology) at 35°C for 3 days. Allantoic fluid was collected, and the virus titer was determined via plaque assay in MDCK cells. All of these procedures were performed in a BSL-2 laboratory.

**Mice**

*Ninj1*^-/-^ (T034357), *Zbp1*^-/-^ (T029037), *Pycard*^-/-^ (T011758), and *Il1b*^-/-^ (T003746) mice on a C57BL/6J background were purchased from GemPharmatech. *Mlkl*^-/-^ (S-KO-14468) and *Nlrp3*^-/-^ (S-KO-05210) mice on a C57BL/6N background were purchased from Cyagen Biosciences. *Gsdmd*^-/-^, *Gsdme*^-/-^, *Gsdmd*^-/-^*Gsdme*^-/-^, *Casp1*^-/-^, and *Il1r*^-/-^ mice on a C57BL/6N background were kind gifts from Dr. Feng Shao from the National Institute of Biological Sciences, Beijing, China. *Gsdmd*^-/-^*Mlkl*^-/-^, *Gsdme*^-/-^*Mlkl*^-/-^, and *Gsdmd*^-/^*Gsdme*^-/-^*Mlkl*^-/-^ mice were obtained by crossing *Gsdmd*^-/-^, *Gsdme*^-/-^, and *Mlkl*^-/-^ mice at our in-house facility. *Ninj1*^-/-^*Il1b*^-/-^ mice were obtained by crossing *Ninj1*^-/-^ and *Il1b*^-/-^ mice at our in-house facility. Wild-type C57BL/6J (N000013) mice were purchased from GemPharmatech, and wild-type C57BL/6N (213) mice were purchased from Beijing Vital River Laboratory Animal Technology. All the mice were kept in a specific pathogen-free facility at the Institute of Biophysics of Chinese Academy of Sciences and maintained on a standard chow diet with a 12-h light/dark cycle. In vivo IAV infection was conducted in the ABSL-2 facility. Male and female mice aged 8-10 weeks were used for IAV challenge. Age- and sex-matched co-housed wild-type mice on the same background served as controls in the animal studies unless otherwise mentioned. All mouse experiments were carried out following the national guidelines for the housing and care of laboratory animals (Ministry of Health, China), and all protocols were reviewed and approved by the Animal Care and Research Committee of the Institute of Biophysics of the Chinese Academy of Sciences.

**Cell line**

THP-1 cells (ATCC, TIB-202™) were maintained in RPMI 1640 (Gibco, A1049101) supplemented with 1X 2-mercaptoethanol (Gibco, 21985023). MDKCs (ATCC, CCL-34) and iBMDMs (a kind gift from Dr. Feng Shao) were maintained in DMEM (Gibco, 31053028). A549 cells were maintained in F-12K medium (ATCC, 30–2004). *Ninj1*^-/-^ iBMDMs, *Ninj1*^-/-^ THP-1 cells and *Ninj1*^-/-^ A549 cells were generated via the CRISPR-Cas9 genome editing system. All culture media were supplemented with 10% FBS (Gibco, A5669701), 100 U/mL penicillin and 100 μg/mL streptomycin (Gibco, 15140122).

**Calculation of the percentage of PI-positive cells**

At the indicated times post infection, 1 μg/mL propidium iodide (PI; Invitrogen, P3566) and 0.2 μg/mL Hoechst 33342 (Invitrogen, H3570) were added to the culture supernatants. Dead cells with plasma membrane leakage were stained with PI, whereas the nuclei of all the cells were stained with Hoechst 33342. Fluorescence images were photographed from 9 randomly selected fields (3 fields/well, 3 wells/group) in each group. The number of cells stained with PI and Hoechst 33342 were counted via ImageJ. The percentage of PI-positive cells was calculated as the ratio of PI-stained cells to Hoechst 33342-stained cells.

**Pathological scores of IAV-induced lung injuries**

The H&E-stained slides were evaluated by two independent pathologists blinded to the experimental groups, with the average score used as the final result. Lung injuries were assessed using a semi-quantitative scoring system that evaluated four key dimensions: alveolar wall thickening, inflammatory cell infiltration, hemorrhage, and perivascular edema. Each dimension was scored independently on a scale of 0-4 as follows: 0, Absent - No detectable abnormalities; 1, Minimal - Changes just beyond the normal range (slight increase); 2, Mild - Observable lesions, not severe (moderate involvement); 3, Moderate - Marked lesions that are likely exacerbated (extensive involvement); 4, Severe - Very pronounced lesions diffusely affecting the entire tissue section. The scores for all four dimensions were summed to yield a composite lung injury score for each sample, with a maximum possible score of 16. Higher scores indicate more severe lung injury.

**Immunofluorescence staining of murine lungs**

Lung sections were deparaffinized and rehydrated. After antigen retrieval, the slides were permeabilized in 0.2% Triton X-100 and then blocked in 5% normal goat serum, followed by overnight incubation with anti-ZO-1 antibodies (Invitrogen, 61-7300, 1:200). Then slides were incubated with Goat anti-Rabbit IgG Secondary Antibody, Alexa Fluor™ Plus 488 (Invitrogen, A48282, 1:500), for 1 hour at room temperature. The slides were subsequently mounted with ProLong™ Gold Antifade Mountant with DAPI (Invitrogen, P36941). For each slide, representative immunofluorescence images were taken at the most severe lesion area. The mean fluorescence intensity (MFI) of ZO-1 was quantified via ImageJ.

**Human BALF sample collection**

BALF samples were collected from 10 hospitalized patients infected with the SARS-CoV-2 Omicron variant and 5 controls. This study was approved by the Ethics Committee of China-Japan Friendship Hospital (2022-KY-058) and was conducted according to the principles expressed in the Declaration of Helsinki. Written informed consents were provided by all study participants or legal representatives for participation in this study. Detailed information on the subjects is listed in Table S2. BALF was filtered through a 70 μm cell filter (Miltenyi, 130-098-462) and centrifuged at 300 × g and 4°C for 5 min to collect the cell precipitates. Cell counts and viability were estimated via a fluorescence cell analyzer (Countstar^®^ Rigel S2) with AO/PI reagent after the removal of erythrocytes (Solarbio, R1010), after which dead cell removal was performed (Miltenyi, 130-090-101). Finally, the fresh cells were washed twice in RPMI 1640 (Gibco, 11875119) and then resuspended at 1×10^6^ cells/ml in RPMI 1640 and 2% FBS (Gibco, 10100147C).

**Single-cell RNA-seq library construction and sequencing**

Single-cell RNA-Seq libraries were prepared via a SeekOne^®^ Digital Droplet Single Cell 3’ library preparation kit (SeekGene, K00202). Briefly, an appropriate number of cells were mixed with reverse transcription reagent and then added to the sample well in SeekOne^®^ chip S3. Barcoded Hydrogel Beads (BHBs) and partitioning oil were subsequently dispensed into the corresponding wells separately in chip S3. After emulsion droplet generation, reverse transcription was performed at 42°C for 90 minutes, followed by enzyme inactivation at 85°C for 5 minutes. The emulsion was then broken to recover cDNA, which was purified and amplified by PCR. The amplified cDNA product was then cleaned, fragmented, end-repaired, A-tailed and ligated to Illumina adapters. Finally, index PCR was performed to enrich sequencing libraries containing the 3' poly-A transcripts, cell barcodes, and unique molecular identifiers (UMIs). The indexed sequencing libraries were cleaned with VAHTS DNA Clean Beads (Vazyme, N411-01), quantified with a Qubit Fluorometer (Thermo Scientific, Q33226), and assessed for size distribution on a Bio-Fragment Analyzer (Bioptic, Qsep400). Sequencing was performed on an Illumina NovaSeq 6000 with a PE150 read length.

**Analysis of scRNA-seq data**

The raw counts of BALF samples were obtained from the raw reads of scRNA-seq aligned with the human GRCh38 reference genome via SeekSoul®Tools. The merged raw count matrix underwent quality control and doublet detection via OmicVerse v1.6.7. After removing low-quality cells and predicted doublets, the remaining raw counts were analyzed for feature selection, dimensionality reduction, nearest neighbor graph construction and visualization, clustering and annotation through Scanpy v1.10.3^2^. Batch correction was performed with BBKNN v1.5.1^3^. The cells were annotated by classical markers provided by Scanpy as follows: ATI/ATII, type I and II alveolar epithelial cells (HOPX, SFTPB); BC, basal cells (KRT13); CC, ciliated cells (FOXJ1); Mo/Ma, monocytes and macrophages (CD14,SPP1); AM, alveolar macrophages (APOE, FABP4); DC, dendritic cells (CLEC9A); Neu, neutrophils (CXCR2); MC, mast cells (TPSB2); CD4T, CD4^+^ T cells (CD3D, CD4); CD8T, CD8^+^ T cells (CD3D, CD8A); NK, natural killer cells (KLRF1); B & PC, B cells & plasma cells (CD79A); Un, Undefined. Gene set scoring was computed with scanpy.tl.score_genes (Hallmark gene sets, MSigDB). Correlation analysis between scores and target genes used Pearson's r. The public COVID-19 snRNA-seq dataset^4^ and the murine IAV lung scRNA-seq dataset^5^ were processed identically. Murine data were aligned to GRCm38/mm10 and annotated as follows: AEC, alveolar epithelial cells (Epcam, Wfdc2); ENDO, endothelial cells (Pecam1, Cdh5); MES, mesenchymal cells (Col1a1, Col3a1); MYE, myeloid cells (S100a8, C1qb); NKT, NK cells and T cells (Cd3d, Nkg7); MO_CLS, classic monocytes (Cd14, Vcan); MO_NCLS, non-classic monocytes (Spn, Ace); MA, macrophages (Apoe, Mafb); AM, alveolar macrophages (Itgax, Siglecf); DC, dendritic cells (Cst3, H2-Ab1); NEU, neutrophils (S100a9, Cxcr2); CD4T, CD4^+^ T cells (Cd3d, Cd3e, Cd4); CD8T, CD8^+^ T cells (Cd3d, Cd3e, Cd8); GDT, γδ T cells (Cd3d, Cd3e, Tmem176a, Tmem176b); ILC2, group 2 innate lymphoid cells (Ccdc184, Kit); NK, natural killer cells (Nrc1, Krb1a); BPC, B cells and plasma cells (Cd79a, Cd79b);

**RNA-seq of IAV-infected BMDMs**

Total RNA was extracted from BMDMs infected with PR8 or mock infected for 12 hours via TRIzol (Invitrogen, 15596018CN). Each group contained 3 biological replicates. The total amount and integrity of the RNA were assessed via the RNA Nano 6000 Assay Kit of the Bioanalyzer 2100 system (Agilent Technologies, CA, USA). Library preparation and RNA-seq via the Illumina NovaSeq 6000 platform were accomplished by Novogene (Beijing, China).

**Bulk RNA-seq data analysis**

Raw reads (FASTQ files) were aligned to the GRCm38/mm10 genome using HISAT2 v2.0.5. Gene-level quantification was performed with featureCounts v1.5.0-p3, followed by FPKM normalization based on gene length and mapped read counts. Differential expression analysis between groups used DESeq2 v1.20.0 with default parameters. Adjusted P-values were calculated via the Benjamini-Hochberg (FDR) procedure. Genes with |log₂(fold change)| > 1 and FDR < 0.05 were deemed significantly differentially expressed. Results were visualized as volcano plots.


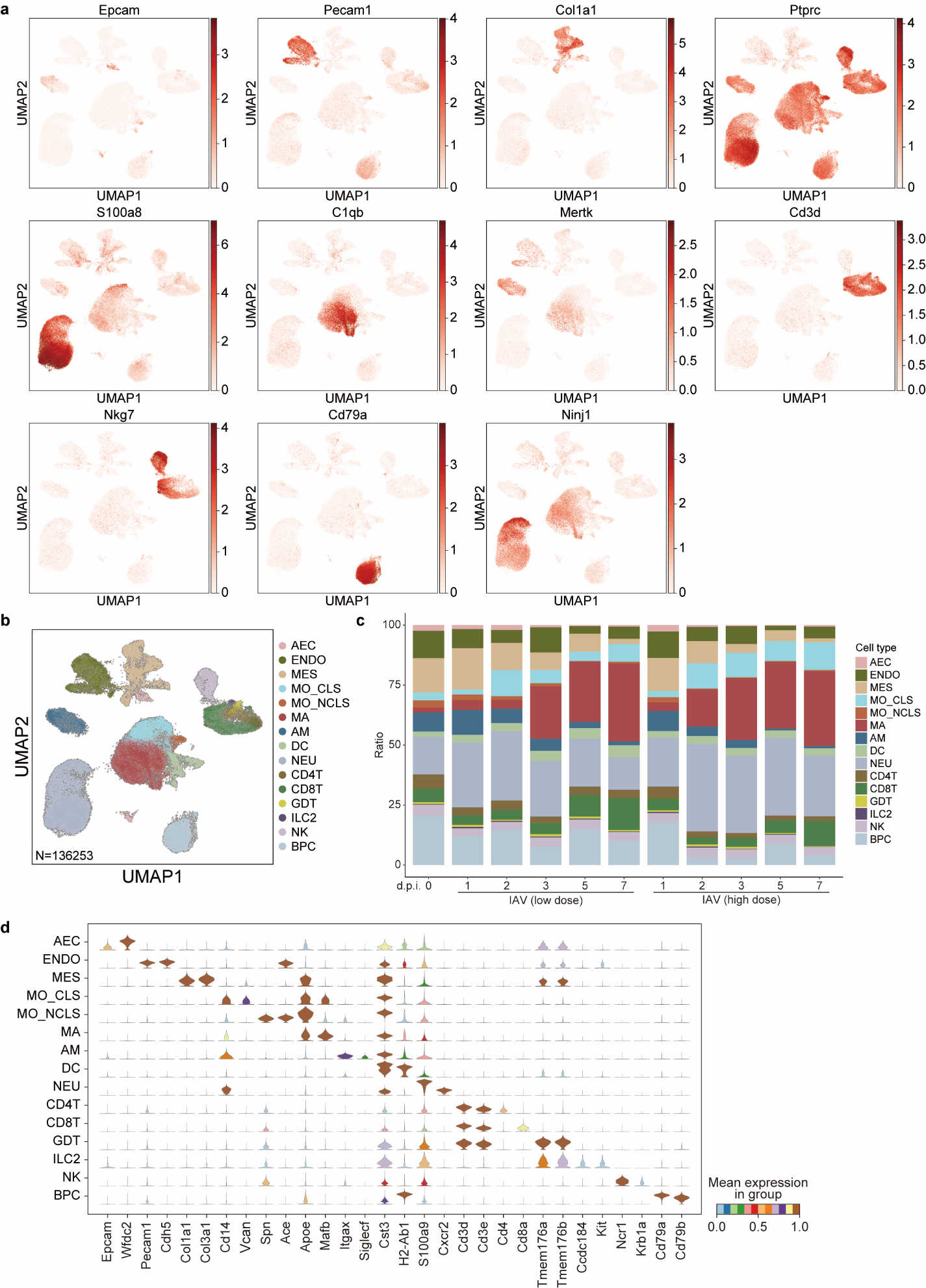


**Figure S1. scRNA-seq analysis of IAV-infected murine lungs**

**a** Expression of hallmark markers for each major cell type on the UMAP embedding (identical to Fig. 1c).

**b** Subcluster resolution of cell subtypes on the UMAP embedding (identical to Fig. 1c).

**c** Proportional composition of cell subtypes across time points and infection groups.

**d** Violin plots depicting expression patterns of hallmark markers per cell subtype.


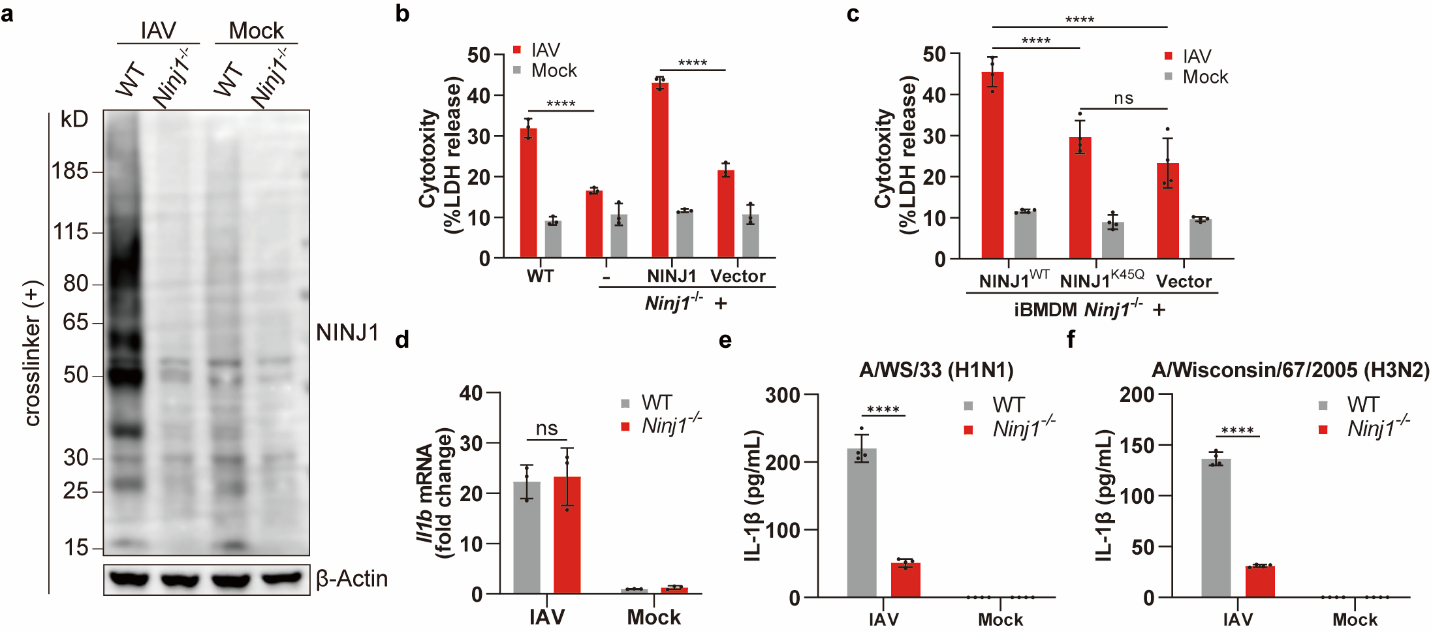


**Figure S2. NINJ1 mediates IAV-induced LDH and IL-1β release**

**a** Immunoblot analysis of NINJ1 in WT and *Ninj1*^-/-^ BMDMs at 12 hpi, BS3-crosslinked.

**b**, **c** LDH release at 12 hpi in WT and *Ninj1*^-/-^ iBMDMs (**b**) as well as *Ninj1*^-/-^ iBMDMs re-expressing NINJ1^WT^ and NINJ1^K45Q^ (**c**).

**d** *Il1b* mRNA expression by qRT-PCR in WT and *Ninj1*^-/-^ BMDMs in Fig. 3h (normalized to uninfected controls).

**e**, **f** IL-1β concentrations in supernatants of BMDMs infected with indicated IAV strains at 16 hpi.

Data are representative of three independent experiments and presented as mean ± SD. Analysis was performed via two-way ANOVA. ns, not significant; *****p* < 0.0001.


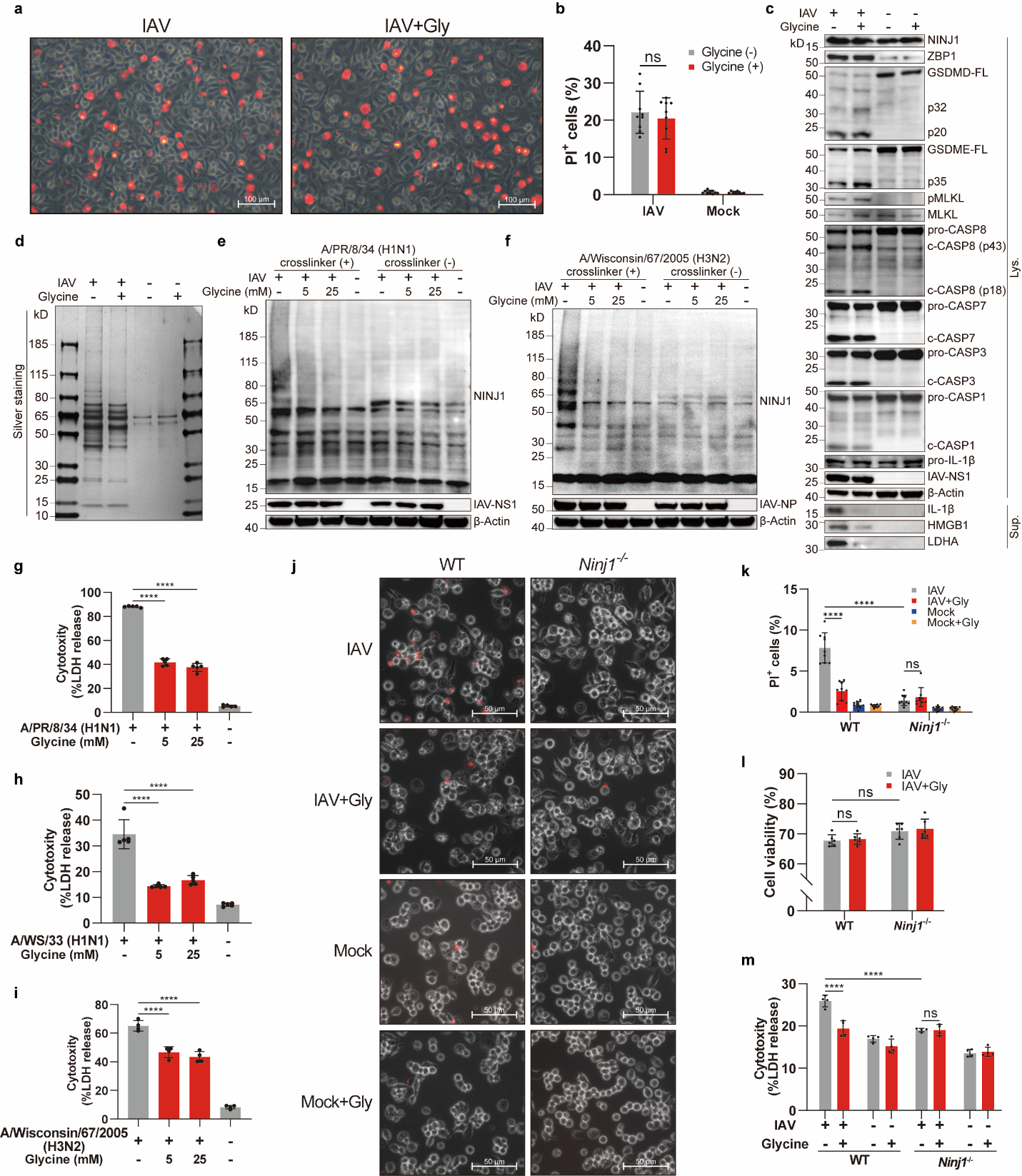


**Figure S3. Glycine treatment inhibits IAV-induced NINJ1 oligomerization and cell lysis**

**a**, **b** Representative images (**a**) and quantification (**b**) of PI^+^ cells at 12 hpi with/without glycine (10 mM). Scale bars, 100 μm.

**c**, **d** Immunoblots of indicated proteins (**c**) in BMDMs with/without glycine (10 mM) at 12 hpi and silver staining of supernatants (**d**).

**e**-**i** Immunoblots of BS3-crosslinked/non-crosslinked NINJ1 (**e**, **f**) and LDH release (**g**-**i**) in BMDMs infected with indicated IAV strains with/without glycine (graded concentrations) at 16 hpi.

**j-m** Representative images (**j**) and quantification (**k**) of PI^+^ cells, cell viability (**l**), and LDH release (**m**) in PMA-differentiated WT and *Ninj1*^-/-^ THP-1 cells with/without glycine (10 mM) at 12 hpi. Scale bars, 50 μm.

Data are representative of three independent experiments and presented as mean ± SD. Analysis was performed via two-way ANOVA (**b**, **k**-**m**) or one-way ANOVA (**g**-**i**). ns, not significant; *****p* < 0.0001.


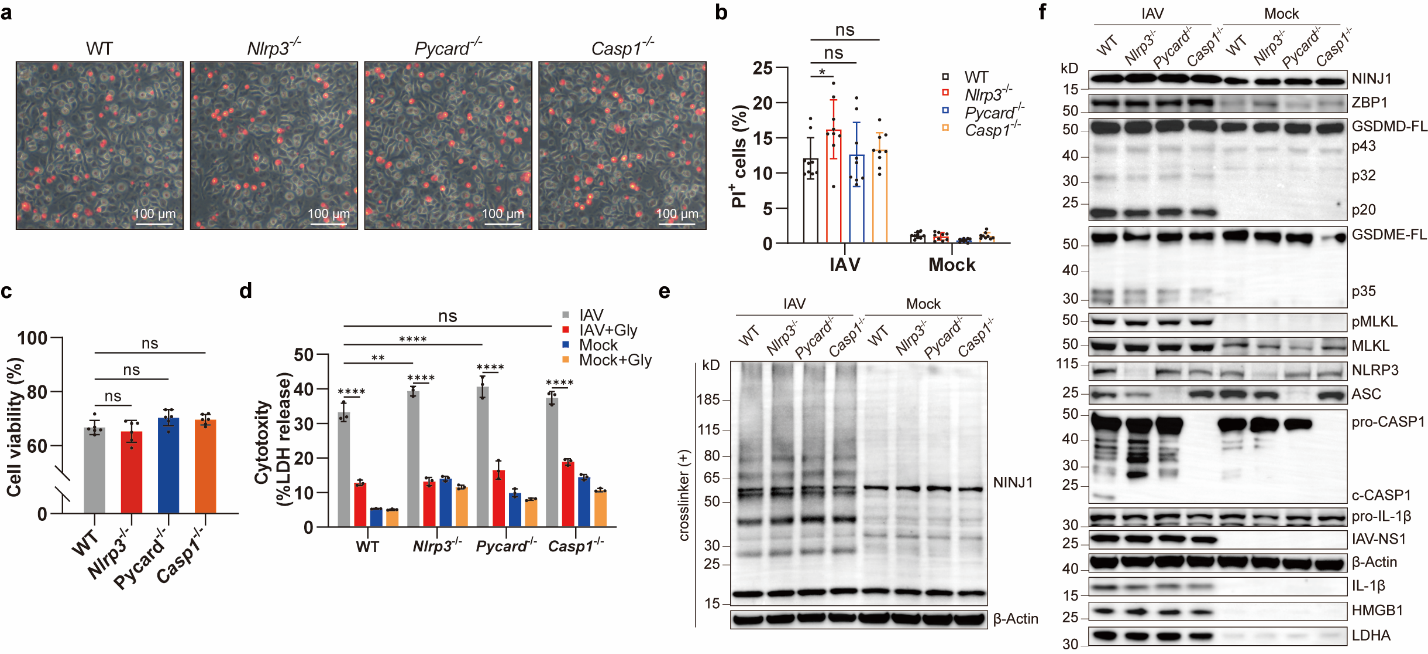


**Figure S4. NINJ1 oligomerization is not regulated by NLRP3 inflammasome**

**a**, **b** Representative images (**a**) and quantification (**b**) of PI^+^ cells, and cell viability (**c**) in WT, *Nlrp3*^-/-^, *Pycard*^-/-^, and *Casp1*^-/-^ BMDMs at 12 hpi. Scale bars, 100 μm.

**d** LDH release in BMDMs (genotypes as above) with/without glycine (10 mM) at 12 hpi.

**e**, **f** Immunoblots of indicated proteins in BMDMs (genotypes as above) at 12 hpi, BS3-crosslinked (**e**) or non-crosslinked (**f**).

Data are representative of three independent experiments and presented as mean ± SD. Analysis was performed via two-way ANOVA (**b**, **d**) or one-way ANOVA (**c**). ns, not significant; **p* < 0.05; ***p* < 0.01; *****p* < 0.0001.


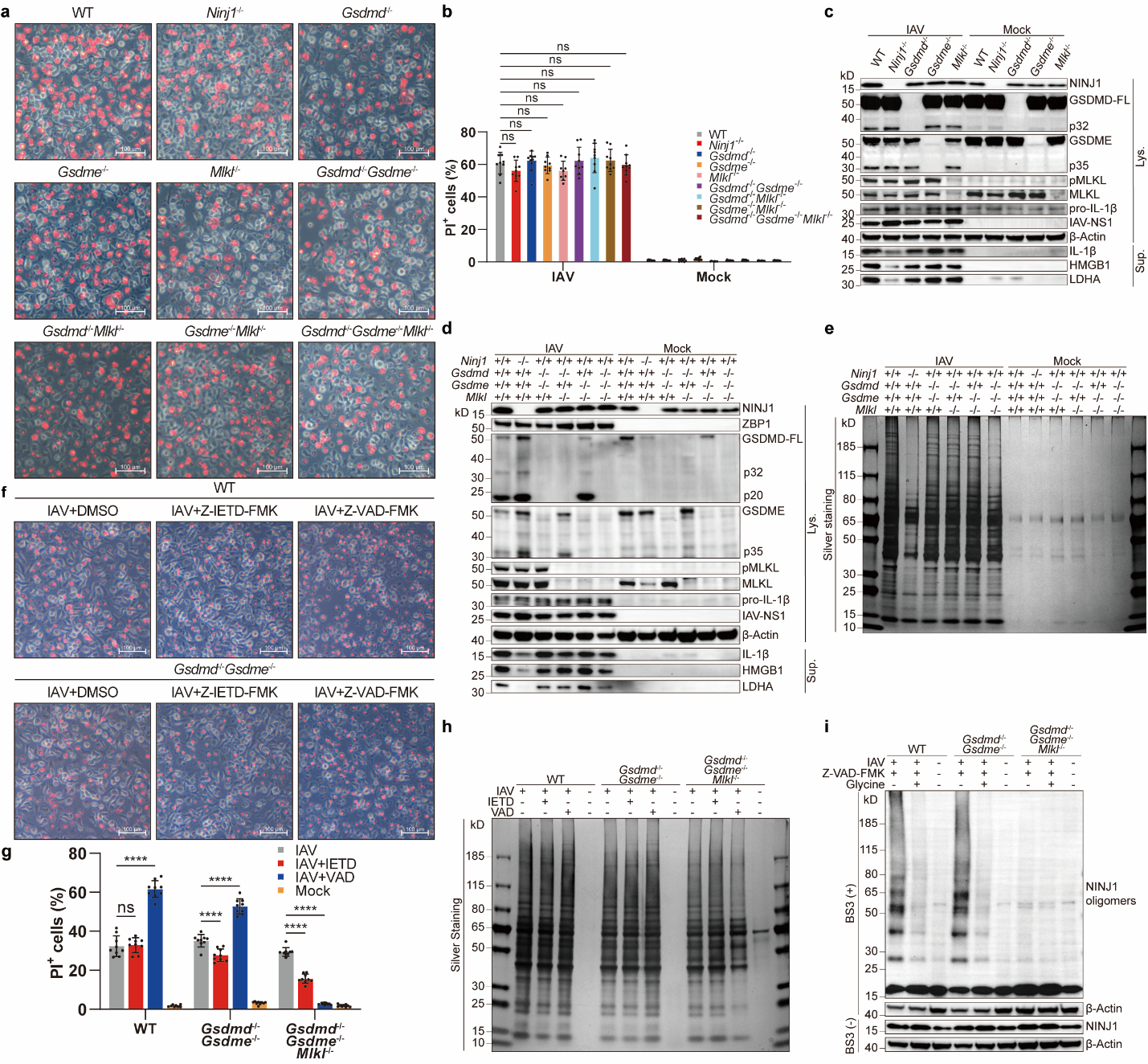


**Figure S5. Activation of any PANoptosis pathway induces NINJ1 oligomerization during IAV infection**

**a**, **b** Representative images (**a**) and quantification (**b**) of PI^+^ cells in BMDMs of indicated genotypes at 16 hpi. Scale bars, 100 μm.

**c**, **d** Immunoblots of indicated proteins in BMDMs (genotypes as above) at 16 hpi.

**e** Silver staining of supernatants from (**d**).

**f**, **g** Representative images (**f**) and quantification (**g**) of PI^+^ cells in WT, *Gsdmd*^-/-^*Gsdme*^-/-^, and *Gsdmd*^-/-^*Gsdme*^-/-^*Mlkl*^-/-^ BMDMs (**f**, related to Fig. 5f) at 12 hpi, treated with DMSO, Z-IETD-FMK (25 μM), or Z-VAD-FMK (25 μM). Scale bars, 100 μm.

**h** Silver staining of supernatants from Fig. 5e.

**i** Immunoblots of BS3-crosslinked NINJ1 in BMDMs with indicated treatments/genotypes at 16 hpi.

Data are representative of three independent experiments and presented as mean ± SD. Analysis was performed using two-way ANOVA. ns, not significant; *****p* < 0.0001.


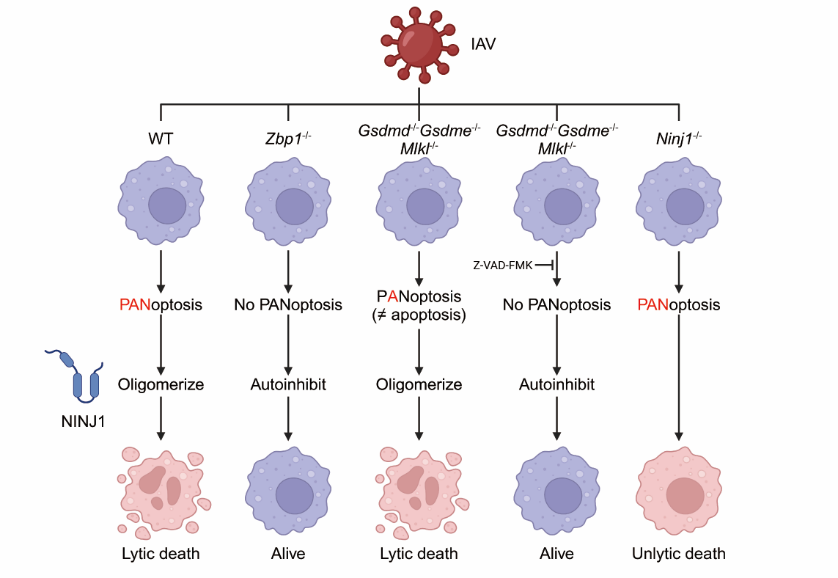


**Figure S6. Schematic of NINJ1 oligomerization coupling to PANoptosis activation in IAV-infected BMDMs**


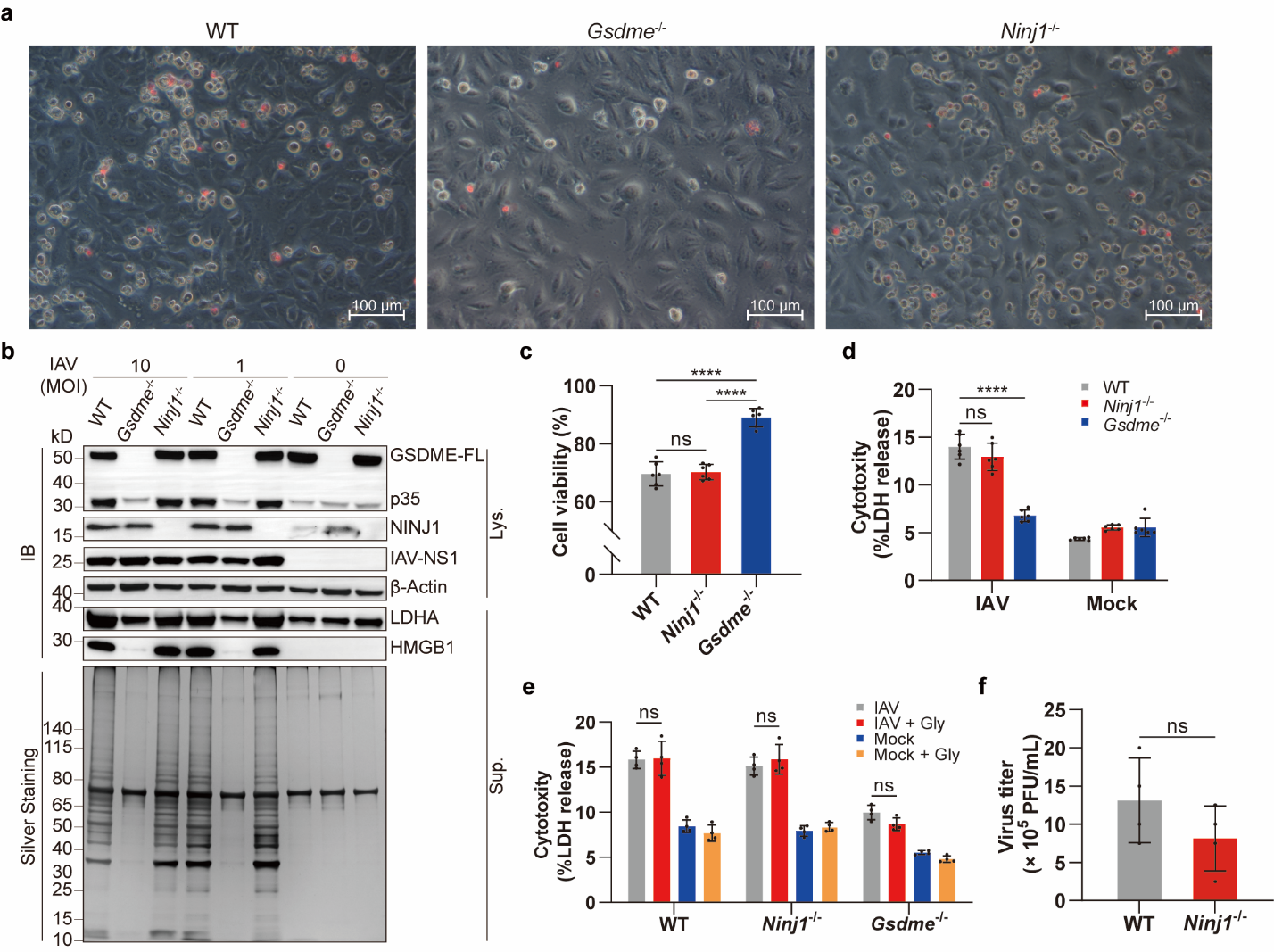


**Figure S7. NINJ1 does not mediate IAV-induced cell lysis in alveolar epithelial cells**

**a** Representative images of PI^+^ cells in WT, *Gsdme*^-/-^, and *Ninj1*^-/-^ A549 cells at 18 hpi. Scale bars, 100 μm.

**b** Immunoblots of indicated proteins and silver staining of supernatants in A549 cells (genotypes as above) at 18 hpi (MOI=1/10).

**c**-**e** Cell viability (**c**) and LDH release (**d**, **e**) in A549 cells (genotypes as above) at 18 hpi, with (**e**)/without (**c**, **d**) glycine (10 mM).

**f** IAV titers in supernatants from WT and *Ninj1*^-/-^ A549 cells at 24 hpi.

Data are representative of three independent experiments and presented as mean ± SD. Analysis was performed via two-way ANOVA (**d**, **e**), one-way ANOVA (**c**), or Student’s t test (**f**). ns, not significant; *****p* < 0.0001.


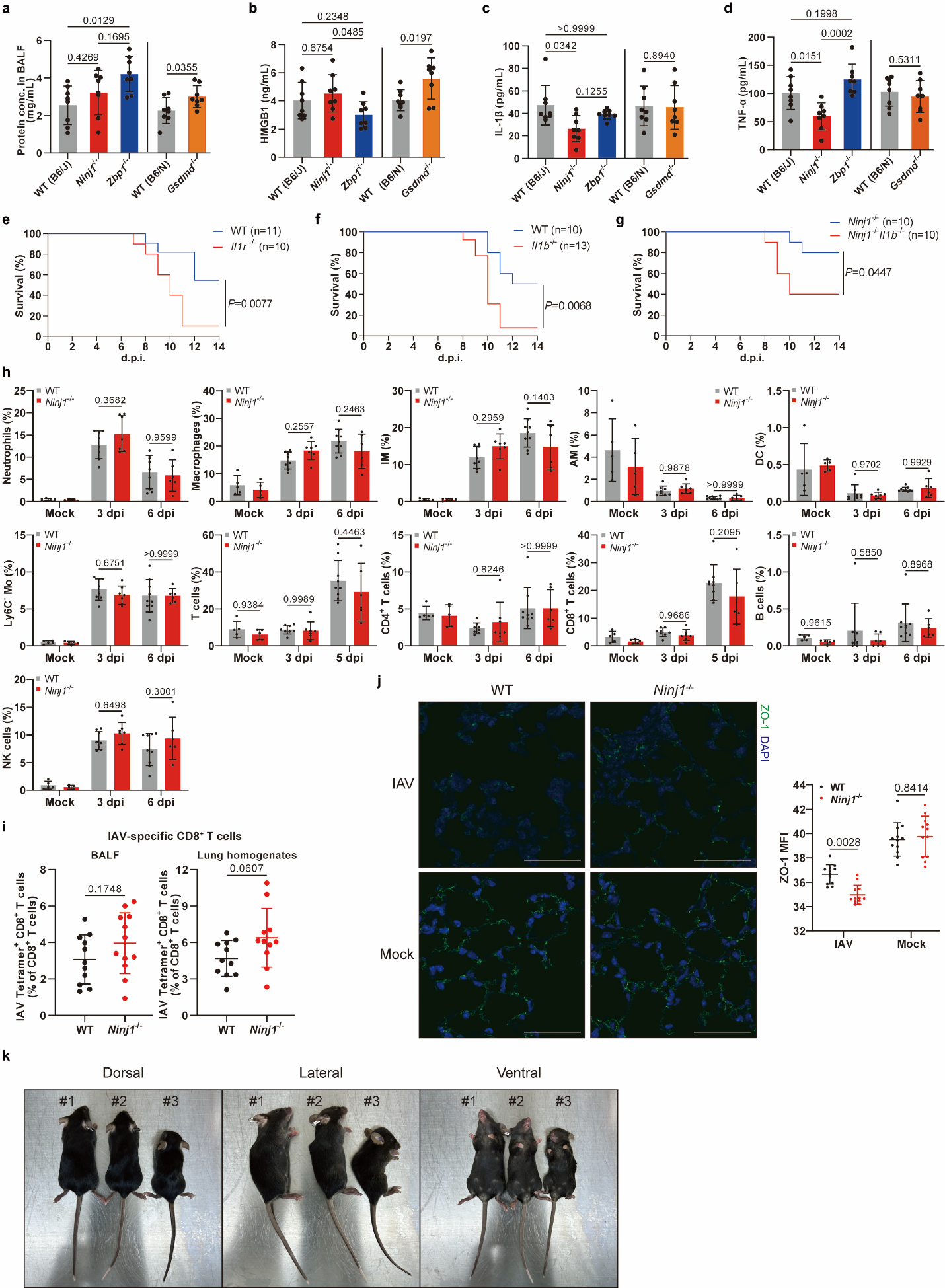


**Figure S8. NINJ1 drives IAV-induced lung pathology and hyperinflammation**

**a**-**d** Concentrations of total proteins (**a**), HMGB1 (**b**), IL-1β (**c**), and TNF-α (**d**) in BALF of WT (C57BL/6J), *Ninj1*^-/-^, *Zbp1*^-/-^, WT (C57BL/6N), and *Gsdmd*^-/-^ mice at 5 dpi (LD_50_ of IAV).

**e**-**g** Survival curves of WT and *Il1r*^-/-^ mice (**e**), WT and *Il1b*^-/-^ mice (**f**), as well as *Ninj1*^-/-^ and *Ninj1*^-/-^*Il1b*^-/-^ mice (**g**) (LD_50_ of IAV).

**h** Frequencies of neutrophils, macrophages, IM (interstitial macrophages), AM (alveolar macrophages), DC (dendritic cells), Ly6C^-^ monocytes (Ly6C^-^ Mo), T cells, CD4^+^ T cells, CD8^+^ T cells, B cells, and NK cells in total live cells in BALF of WT and *Ninj1*^-/-^ mice at 3 and 6 dpi (LD_50_ of IAV). Gating strategies were shown in Supplementary Figs. 9a, 9b.

**i** Frequencies of IAV-specific CD8^+^ T cells (H-2D^b^-restricted influenza NP-specific CD8^+^ T cells, influenza NP_366-374_ peptide) in total CD8^+^ T cells in BALF of WT and *Ninj1*^-/-^ mice at 8 dpi (LD_50_ of IAV). Gating strategies were shown in Supplementary Fig. 9c.

**j** Immunofluorescence images of ZO-1 in lung sections from WT and *Ninj1*^-/-^ mice (LD_50_ of IAV). Scale bars, 50 μm. Mean Fluorescence Intensity (MFI) of ZO-1 were quantified.

**k** Phenotypes of 8-week-old female mice: #1, WT, normal; #2, *Ninj1*^-/-^, normal; #3, *Ninj1*^-/-^, stunted growth, hydrocephaly, ataxia.

Data are representative of at least two independent experiments and presented as mean ± SD. Kruskal-Wallis test (**c**) or one-way ANOVA (**a**, **b**, **d**) was used in comparisons among WT, *Ninj1*^-/-^ , and *Zbp1*^-/-^ groups. Student’s t test was used in (**i**) and in comparisons between WT and *Gsdmd*^-/-^ groups (**a**-**d**). Two-way ANOVA was applied in (**h**, **j**). Survival curves were analyzed via log-rank test (**e**-**g**).


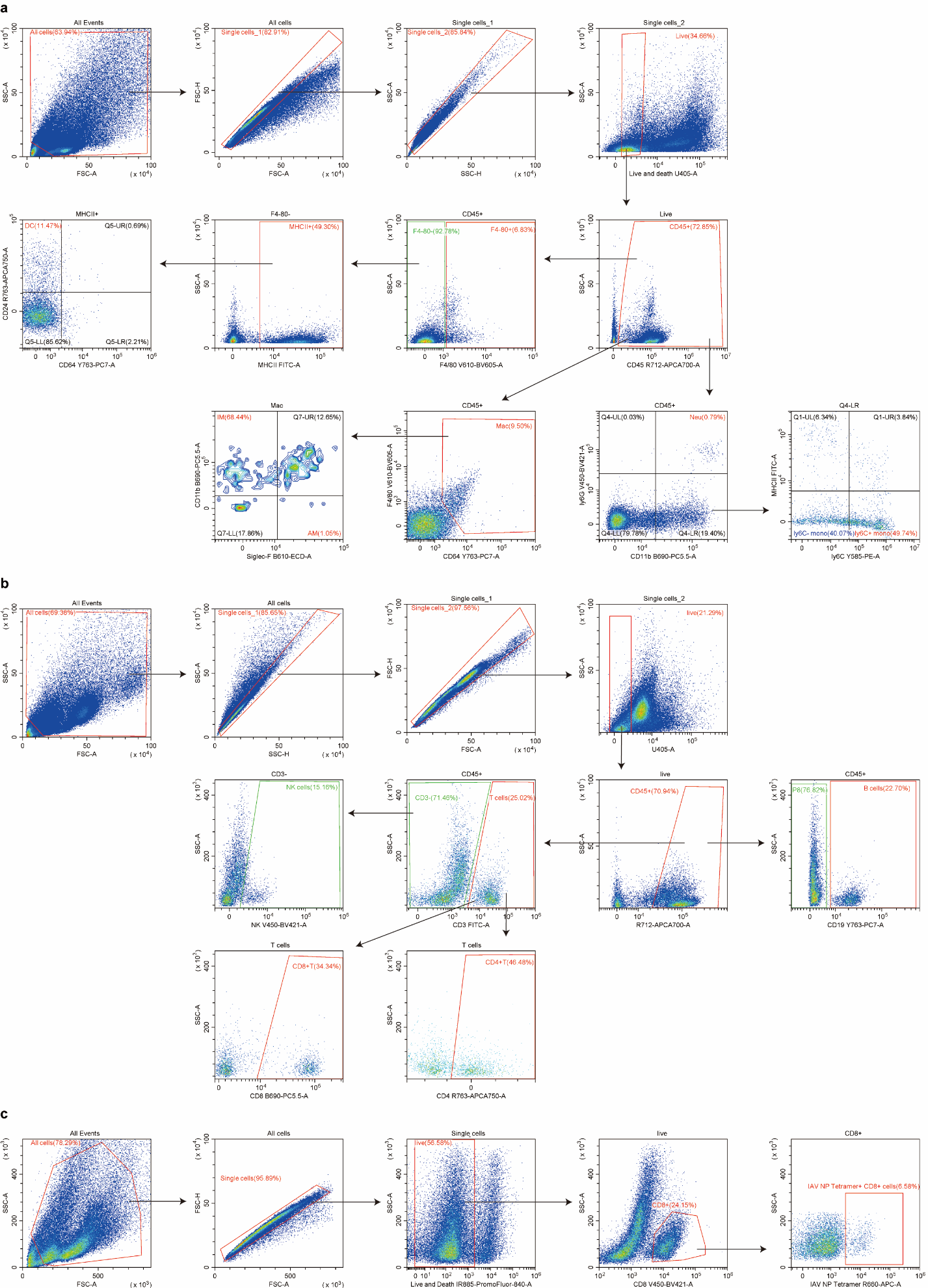


**Figure S9. Gating Strategies for flow cytometric analyses**

**a**, **b** Gating strategies for Figs. 6m, 6n, and Supplementary Fig. 8h. Cell types were defined as followed: macrophages (Mac), CD45^+^CD64^+^F4/80^+^ cells; interstitial macrophages (IM), CD11b^+^Siglec-F^-^ macrophages; alveolar macrophages (AM), CD11b^-^Siglec-F^+^ macrophages; Ly6C^+^ monocyte (Ly6C^+^ mono), CD45^+^CD11b^+^Ly6G^-^MHC-II^-^Ly6C^+^ cells; Ly6C^-^ monocyte (Ly6C^-^mono), CD45^+^CD11b^+^Ly6G^-^MHC-II^-^Ly6C^-^ cells; dendritic cells (DC), CD45^+^F4/80^-^MHC-II^+^CD64^-^CD24^+^ cells; T cells, CD45^+^CD3^+^ cells; natural killer cells (NK cells), CD45^+^CD3^-^NK1.1^+^ cells; B cells, CD45^+^CD19^+^ cells

**c** Gating strategies for Supplementary Fig. 8i. IAV-specific CD8^+^ T cells were defined as CD8^+^ H-2D^b^ Influenza NP Tetramer^+^ cells.


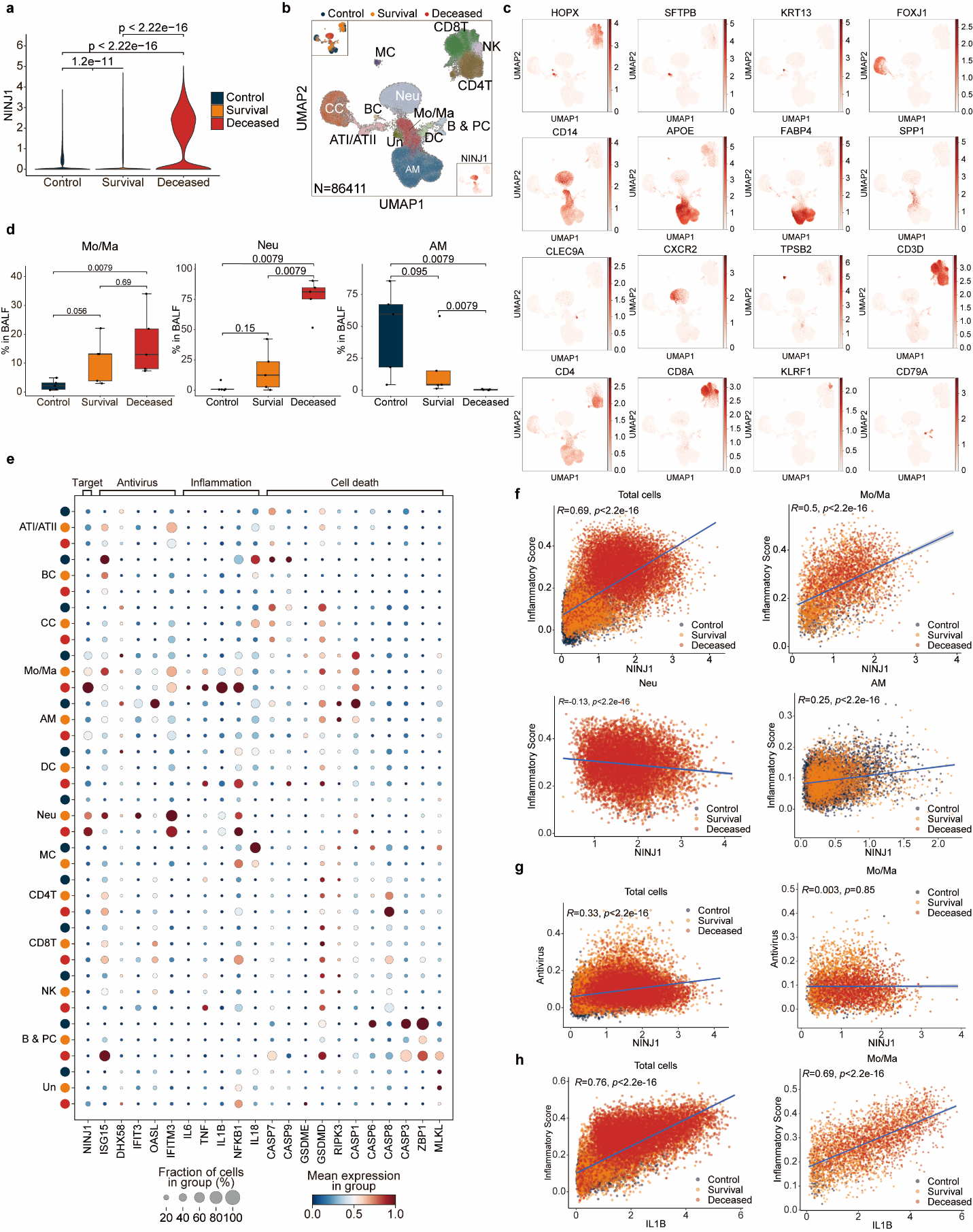


**Figure S10. NINJ1 is associated with hyperinflammation and poor outcome in patients with COVID-19**

**a** NINJ1 expression levels across three groups (violin plot).

**b** UMAP embedding of 86,411 single cells from 15 BALF samples. ATI/ATII, type I and II alveolar epithelial cell; BC, basal cell; CC, ciliated cell; Mo/Ma, monocyte and macrophage; AM, alveolar macrophage; DC, dendritic cell; Neu, neutrophil; MC, mast cell; CD4T, CD4^+^ T cell; CD8T, CD8^+^ T cell; NK, natural killer cell; B & PC, B cell & plasma cell; Un, Undefined.

**c** Hallmark marker expression per cell type from (**b)**

**d** Proportional changes in Mo/Mas (left), Neus (middle) and AMs (right) among groups.

**e** Dot plot of NINJ1 and indicated genes' expression patterns per cell type across groups.

**f** Correlation of NINJ1’s expression level and inflammatory score in total cells (upper left), Mo/Mas(upper right), Neus (lower left), and AMs (lower right).

**g** Correlation of NINJ1’s expression level and antivirus score in total cells (left) and Mo/Mas (right).

**h** Correlation of IL1B’s expression level and inflammatory score in total cells (left) and Mo/Mas (right).


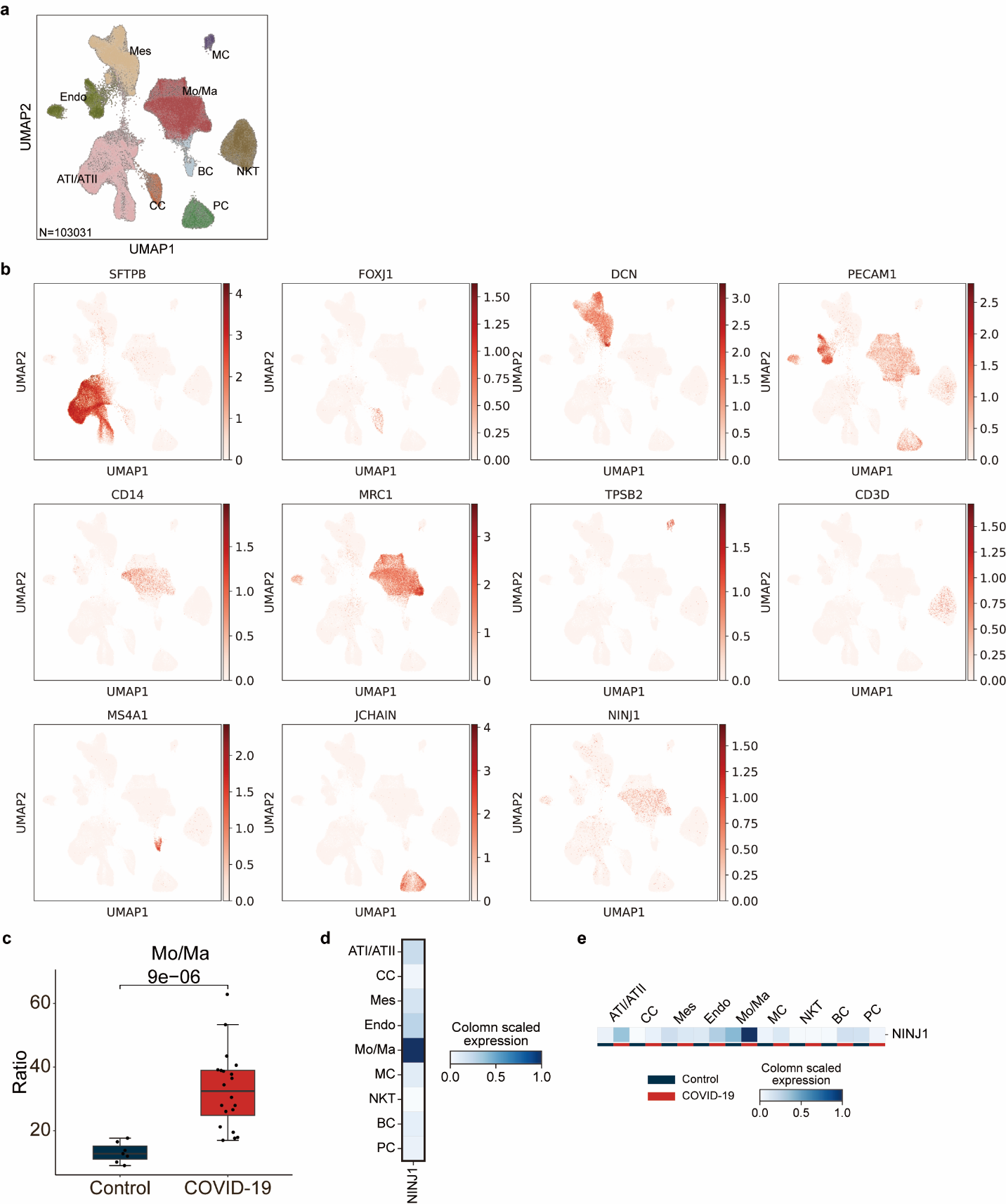


**Figure S11. scRNA-seq analysis of a published dataset GSE171524**

**a** UMAP clustering with cell-type annotations (ATI/ATII, type I and II alveolar epithelial cell; Mes, mesenchymal cell; Endo, endothelial cells Mo/Ma, monocyte and macrophage; CC, ciliated cell; MC, mast cell; NKT, natural killer cell and T cell; BC, B cell; PC, plasma cell).

b Hallmark marker scores per cell type overlaid on UMAP from (**a**).

**c** Mo/Ma proportion comparison between clinical groups.

**d** Matrix plot: NINJ1 expression levels across cell types.

**e** Matrix plot: NINJ1 expression per cell cluster stratified by clinical groups.

**Table S3. Antibodies used in immunoblots**

| Antibodies | Source | Identifier |
| --- | --- | --- |
| Rabbit polyclonal anti-NINJ1 | ABclonal | Cat# A16406; RRID:AB_2770588 |
| Rabbit monoclonal anti-GSDMD (mouse) | Abcam | Cat# ab209845; RRID:AB_2783550 |
| Rabbit monoclonal anti-GSDME | Abcam | Cat# ab215191; RRID:AB_2737000 |
| Rabbit monoclonal Anti-MLKL (phospho S345, mouse) | Abcam | Cat# ab196436; RRID:AB_2687465 |
| Rabbit polyclonal Anti-MLKL (mouse) | abcepta | Cat# AP14272b; RRID:AB_11134649 |
| Mouse monoclonal anti-ZBP1 | AdipoGen | Cat# AG-20B-0010; RRID:AB_2490191 |
| Mouse monoclonal anti-Caspase-1 (p20, mouse) | AdipoGen | Cat# AG-20B-0042; RRID:AB_2490248 |
| Rabbit monoclonal anti-Caspase-3 | Cell Signaling Technology | Cat# 14220; RRID:AB_2798429 |
| Rabbit polyclonal anti-Caspase-7 | Cell Signaling Technology | Cat# 9492; RRID:AB_2228313 |
| Rabbit polyclonal anti-Caspase-8 (mouse) | Cell Signaling Technology | Cat# 4927; RRID:AB_2068301 |
| Rabbit monoclonal anti-cleaved Caspase-8 (mouse) | Cell Signaling Technology | Cat# 8592; RRID:AB_10891784 |
| Mouse monoclonal anti-NLRP3 | AdipoGen | Cat# AG-20B-0014; RRID:AB_2490202 |
| Rabbit polyclonal anti-Caspase-9 (mouse) | Cell Signaling Technology | Cat# 9504; RRID:AB_2275591 |
| Rabbit polyclonal anti-cleaved Caspase-9 (mouse) | Cell Signaling Technology | Cat# 9509; RRID:AB_2073476 |
| Rabbit polyclonal anti-ASC | AdipoGen | Cat# AG-25B-0006; RRID:AB_2490440 |
| Rabbit polyclonal Anti-HMGB1 | Abcam | Cat# ab18256, RRID:AB_444360 |
| Rabbit polyclonal Anti-LDHA | Proteintech | Cat# 19987-1-AP; RRID:AB_10646429 |
| Rabbit polyclonal Anti-IL-1β (mouse) | Proteintech | Cat# 26048-1-AP; RRID:AB_2880351 |
| Mouse monoclonal anti-influenza A virus NS1 | Santa Cruz Biotechnology | Cat# sc-130568; RRID:AB_2011757 |
| Rabbit monoclonal anti-influenza A virus NP | Thermo Fisher Scientific | Cat# MA5-42365; RRID:AB_2911506 |
| HRP-conjugated rabbit monoclonal anti-β-Actin | ABclonal | Cat# AC043; RRID:AB_3065551 |
| HRP-conjugated goat polyclonal anti-rabbit IgG | Cell Signaling Technology | Cat# 7074; RRID:AB_2099233 |
| HRP-conjugated goat polyclonal anti-mouse IgG | Cell Signaling Technology | Cat# 7076, RRID:AB_330924 |

**Table S4. CRISPR-Cas9 guide RNA sequences**

| gRNA | Sequences |
| --- | --- |
| m*Ninj1* gRNA-1, iBMDMs | GTGGGGTGTTGCACTGAGCGGGG |
| m*Ninj1* gRNA-2, iBMDMs | ACACGGCATCTCGTCCATGGTGG |
| h*Ninj1* gRNA-1, A549 cells | GGCGTAATGGTTCACGTTGATGG |
| h*Ninj1* gRNA-2, A549 cells | CTTGTGCTGCAGATCGGCGTGGG |
| h*Ninj1* gRNA-3, THP-1 cells | GAGGAGTACGAGCTCAACGGCGG |

**Table S5. Primers for qRT-PCR**

| Primers | Sequences |
| --- | --- |
| m*Ninj1* F | CTGCTCATCTTCCTGGTCAAGT |
| m*Ninj1* R | GCCACGTCCATTACAGGCTT |
| m*Gapdh* F | TGCCCCCATGTTTGTGATG |
| m*Gapdh* R | TGTGGTCATGAGCCCTTCC |
| m*Il1b* F | TGGACCTTCCAGGATGAGGACA |
| m*Il1b* R | GTTCATCTCGGAGCCTGTAGTG |
| IAV-NP F | CAGCCTAATCAGACCAAATG |
| IAV-NP R | TACCTGCTTCTCAGTTCAAG |

**Table S6. Antibodies used in flow cytometry**

| Antibodies | Source | Identifier |
| --- | --- | --- |
| Alexa Fluor® 700 anti-mouse CD45 | BioLegend | Cat# 103128; RRID:AB_493715 |
| Brilliant Violet 421™ anti-mouse Ly-6G (BioLegend,) | BioLegend | Cat# 127628; RRID:AB_256256 |
| PerCP/Cyanine5.5 anti-mouse/human CD11b | BioLegend | Cat# 101228; RRID:AB_893232 |
| PE/Cyanine7 anti-mouse CD64 (FcγRI) | BioLegend | Cat# 139314; RRID:AB_2563904 |
| Brilliant Violet 605™ anti-mouse F4/80 | BioLegend | Cat# 123133; RRID:AB_2562305 |
| PE anti-mouse Ly-6C | BioLegend | Cat# 128008; RRID:AB_1186132 |
| FITC anti-mouse I-A/I-E | BioLegend | Cat# 107606; RRID:AB_313321 |
| PE/Dazzle™ 594 anti-mouse CD170 (Siglec-F) | BioLegend | Cat# 155530; RRID:AB_2890716 |
| FITC anti-mouse CD3 | BioLegend | Cat# 100204; RRID:AB_312661 |
| APC/Cyanine7 anti-mouse CD4 | BioLegend | Cat# 100414; RRID:AB_312699 |
| PerCP/Cyanine5.5 anti-mouse CD8a | BioLegend | Cat# 100734; RRID:AB_2075238 |
| PE/Cyanine7 anti-mouse CD19 | BioLegend | Cat# 115520; RRID:AB_313655 |
| Brilliant Violet 421™ anti-mouse NK-1.1 | BioLegend | Cat# 108732; RRID:AB_2562218 |
| APC/Fire™ 750 anti-mouse CD24 | BioLegend | Cat# 101840; RRID: RRID:AB_2650876 |
| Pacific Blue™ anti-mouse CD8a Antibody | BioLegend | Cat# 100725; RRID:AB_493425 |

**References in Supplementary Materials**

1. Toda, G., Yamauchi, T., Kadowaki, T. & Ueki, K. Preparation and culture of bone marrow-derived macrophages from mice for functional analysis. *STAR Protoc* **2**, 100246 (2021).
2. Wolf, F. A., Angerer, P. & Theis, F. J. SCANPY: large-scale single-cell gene expression data analysis. *Genome Biol* **19**, 15 (2018).
3. Polański, K. *et al.* BBKNN: fast batch alignment of single cell transcriptomes. *Bioinformatics* **36**, 964-965 (2020).
4. Melms, J. C. *et al.* A molecular single-cell lung atlas of lethal COVID-19. *Nature* **595**, 114-119 (2021).
5. Zheng, Y. *et al.* Influenza A virus dissemination and infection leads to tissue resident cell injury and dysfunction in viral sepsis. *EBioMedicine* **116**, 105738 (2025).
